# Genomic features defining exonic variants that modulate splicing

**DOI:** 10.1186/gb-2010-11-2-r20

**Published:** 2010-02-16

**Authors:** Adam Woolfe, James C Mullikin, Laura Elnitski

**Affiliations:** 1 Genomic Functional Analysis Section, National Human Genome Research Institute, National Institutes of Health, Rockville, Maryland 20892, USA; 2 Comparative Genomics Unit, National Human Genome Research Institute, National Institutes of Health, Rockville, Maryland 20892, USA

## Abstract

**Background:**

Single point mutations at both synonymous and non-synonymous positions within exons can have severe effects on gene function through disruption of splicing. Predicting these mutations *in silico *purely from the genomic sequence is difficult due to an incomplete understanding of the multiple factors that may be responsible. In addition, little is known about which computational prediction approaches, such as those involving exonic splicing enhancers and exonic splicing silencers, are most informative.

**Results:**

We assessed the features of single-nucleotide genomic variants verified to cause exon skipping and compared them to a large set of coding SNPs common in the human population, which are likely to have no effect on splicing. Our findings implicate a number of features important for their ability to discriminate splice-affecting variants, including the naturally occurring density of exonic splicing enhancers and exonic splicing silencers of the exon and intronic environment, extensive changes in the number of predicted exonic splicing enhancers and exonic splicing silencers, proximity to the splice junctions and evolutionary constraint of the region surrounding the variant. By extending this approach to additional datasets, we also identified relevant features of variants that cause increased exon inclusion and ectopic splice site activation.

**Conclusions:**

We identified a number of features that have statistically significant representation among exonic variants that modulate splicing. These analyses highlight putative mechanisms responsible for splicing outcome and emphasize the role of features important for exon definition. We developed a web-tool, Skippy, to score coding variants for these relevant splice-modulating features.

## Abstract 2

A comparative analysis of SNPs and their exonic and intronic environments identifies the features predictive of splice affecting variants.

## Background

The majority of genes in mammalian genomes are made up of multiple exons separated by much longer introns. To create a mature mRNA, exons must be identified accurately from within the transcript and then spliced together by removing the intervening introns. This process is carried out by a large complex of small nuclear RNAs and polypeptides known as the spliceosome. Disruption to the fidelity of splicing, particularly of exons that are constitutively spliced, can effectively inactivate a gene by creating unstable mRNAs and defective protein structure, or cause disease by disrupting the balance of expression of different splice isoforms [1]. The most important features for exon recognition are the splice junctions that define the boundaries of the exons, at which the spliceosome must assemble. Mutations at sites causing splicing abnormalities make up around 15% of all point mutations that result in human genetic disease [2]. However, this figure is likely to be a significant underestimate of the contribution of splicing in disease, as there is an increasing number of studies showing that mutations within both exons and introns, but outside of the canonical splice sites, can also disrupt splicing [3]. In particular, the ability of nonsense, missense and even synonymous (silent) mutations to cause exon skipping is often overlooked due to the strong association of exonic mutations solely with protein coding changes. Indeed, as the skipping of the exon can lead to the removal of an entire protein domain or degradation of the mRNA via nonsense-mediated decay, splice-affecting variants (including synonymous changes) are much more deleterious than most missense mutations that substitute a single amino acid. Similarly, exonic variants can also result in deleterious effects by activating a *de novo *(that is, not pre-existing) ectopic splice site, which is then used in preference to the natural splice site, shortening the exon. A well-known example of this is a synonymous mutation in exon 11 of the human *LMNA *gene that creates a 5' ectopic splice site. This shortens the protein sequence through frameshift, and causes the rare premature aging disorder Hutchinson-Gilford progeria [4].

The mechanism by which these internal exonic mutations exert their effect is still not fully understood, but they are most commonly associated with changes in regulatory elements within the exon that are important for exon definition. The spliceosome must distinguish genuine splice sites from a collection of sequences in the intron that resemble them but are never used (known as pseudo splice-sites). Therefore, correct exon recognition requires additional auxiliary signals present both within the exon and in the introns, as canonical splice sites are not sufficient to define the proper splice sites. These regulatory sequences, important in both constitutive and alternative splicing, can be broadly defined by their intergenic location and their effects on splicing. Those located within the exon and promoting exon inclusion are referred to as exonic splicing enhancers (ESEs) and those inhibiting exon inclusion are referred to as exonic splicing silencers (ESSs). Similarly those located in the intron are referred to as intronic splicing enhancers and intronic splicing silencers, although these are more commonly associated with specifying alternative splicing [5] or splicing of non-canonical introns [6].

Although identification and characterization of the complement of proteins that bind specific exonic enhancer and silencer elements is far from complete, most enhancer sequences within exons have been found to bind members of the serine/arginine-rich (SR) protein family, while many silencing elements are bound by members of the heterogeneous ribonuclearprotein (hnRNP) family [7]. ESE-bound SR proteins promote exon definition by directly recruiting and stabilizing the splicing machinery through protein-protein interactions [8] and/or antagonizing the function of nearby silencer elements [9]. Silencers are not as well characterized as enhancers, but ESS-bound hnRNPs are thought to mediate silencing through direct antagonism of the splicing machinery or by direct competition for overlapping enhancer-binding sites. The intrinsic strength by which the splice sites are recognized by the spliceosome as well as the antagonistic dynamics of proteins binding ESEs and ESSs control much of exon recognition and alternative splicing. It is therefore not surprising that exonic splicing regulatory sequences (ESRs) are now increasingly recognized as a major target and a common mechanism for disease-causing mutations leading to exon skipping in functionally diverse genes. Examples of disease mutations reported to destroy ESE motifs and cause exon skipping include those in the *BRCA1 *[10], *SMN1*/*2 *[11], *PDHA1 *[12] and *GH *[13] genes.

Given the critical role of these sequences in exon splicing, significant research efforts are focused on identifying the complement of ESE and ESS binding sites involved in constitutive splicing. The assortment of enhancer and silencer sequences recognized by known splicing factors is considerable [3]. This suggests that ESRs may represent numerous functionally distinct classes, or may be recognized in a degenerate fashion. This 'fuzzy' definition of ESRs has meant that their precise characterization has proved challenging. A large group of existing ESE/ESS datasets has been identified either experimentally [14,15] or through the use of computational approaches followed by some form of experimental verification of a subset of predictions [16-19] (for an overview see Table 1 and [20]). The motifs defined in each dataset are commonly represented as hexamers or octomers, or encoded as position weight matrices analogous to transcription factor binding sites. Motifs predicted by these approaches are partially overlapping, but also yield certain proportions that are unique or even contradictory. Recent studies have also suggested that both global and local RNA secondary structure may also play a role in the recognition and activity of splicing regulatory motifs in certain cases [21,22]. Despite our access to these varied splicing regulatory datasets, the question of whether they are effective in detecting the appropriate splicing regulatory changes associated with splice modulating variants has yet to be systematically assessed.

**Table 1:** Exonic splicing regulatory elements datasets used in this study

ESR dataset	Format	Method	Reference
ESEFinder	4 ESE PWMs	Set of four experimentally derived ESE binding site matrices for four SR proteins (SF2, SC35, SRp40, SRp55) identified by an *in vitro *SELEX approach with specific SR protein complementation	[14]
Fas- (hex3)ESS	176 ESS hexamers	Set of experimentally derived ESSs identified *in vivo *through cloning of random decamers into fluorescence activated minigene reporter by selecting those sequences that cause exon skipping. Unique candidates were clustered and represented by non-degenerate hexamers	[15]
RESCUE-ESE	238 ESE hexamers	Set of putative ESEs derived from overrepresented hexamer motifs in exons versus introns and exons with weak splice sites versus exons with strong splice sites	[17]
PESX	2,096 ESE/974 ESS octomers	Set of putative ESEs (PESE) and ESSs (PESS) overrepresented and underrepresented in internal non-coding exons versus unspliced pseudoexons and 5' UTRs of intronless genes	[18]
NI-ESR	979 ESE/496 ESS hexamers	Uses the neighborhood inference (NI) algorithm to identify new candidate ESEs and ESSs using a set of previously identified ESEs/ESSs. The NI algorithm searches the sequence neighborhood of a particular hexamer and scores it by whether the surrounding sequences contain mostly known ESEs, ESSs or neither. Predicted candidates were verified by cross-validation and a subset was experimentally validated	[19]
Ast-ESR	285 hexamers	Motifs based on computational analysis of overrepresented and conserved dicodons in orthologous human-mouse exons. Putative ESRs are not labeled as ESEs or ESSs as a number were found to act as both enhancers and silencers in minigene assays depending on sequence context.	[16]
Composite-ESR	400 ESE/217 ESS hexamers	Combined set of ESE/ESS based on RESCUE-ESE, PESE, PESS and Fas-ESS datasets	[60]

PWM, position weight matrix.

The development of high throughput sequencing technologies provides an unprecedented opportunity to identify disease alleles associated with both common and rare disorders. In the likelihood that exonic splice-affecting mutations are a commonly overlooked phenomena in disease and transcript variation, it is important to identify the genomic features most relevant in characterizing novel splice-affecting genome variants (SAVs). We performed a comparative analysis using sets of experimentally verified SAVs against SNPs common in the human population, the majority of which are likely to be splicing-neutral. Comparative analysis of SNP datasets is a powerful approach to highlight characteristics that define disruptive sequence variants. A similar approach has been employed previously to predict SNPs affecting transcriptional *cis*-regulation [23] as well as to measure selective pressure on genomic elements such as conserved non-coding sequences [24] and splicing enhancers [25]. Here, we focused our main analyses on the most prevalent and least characterized SAVs, those that cause exon skipping, using a battery of bioinformatics approaches as well as a systematic comparison of all currently available ESE/ESS datasets, to identify the features of these SAVs and their exonic/intronic environment that are most likely to be predictive for exon skipping events. Extending this analysis, we also identified relevant features associated with SAVs causing increased exon inclusion and ectopic splice site creation. Combined, these features are useful to predict the probability of novel splice-modulatory events and are made available through a web server.

## Results

To identify features associated with exon skipping SAVs, we collated a set of experimentally verified variants in the human genome that independently cause exon skipping from extensive literature searches and the Alternative Splicing Mutation Database [26]. We excluded all variants from this list that may affect splicing through other well-defined mechanisms, such as nonsense-mediated exon skipping or disruption of canonical splice sites (see Materials and methods). A total of 87 variants were identified (currently the largest dataset of its kind), and their genomic positions mapped back onto the human genome (hg18). As the majority of analyses in this paper involve exon-skipping SAVs, we refer to these variants simply as 'SAVs', unless otherwise indicated. This set is made up of 32 synonymous and 55 missense SAVs distributed across 43 genes and 47 individual exons (Additional file 1). Of these, 87% (41) were constitutively spliced and 13% (6) were alternatively spliced cassette exons.

In addition to known SAVs, a set of spicing-neutral variants (that is, that have little or no effect on exon splicing) served as a standard for comparison. Although no large-scale set of experimentally verified splice-neutral variants has been published, through literature searches we identified a set of 80 variants that were tested in mini-gene splicing assays and were found to have no effect on splicing (Additional file 2). Unfortunately, around half of these derive from artificial mutagenesis studies, and may therefore include certain artificial biases. As an alternative, since exon-skipping events are likely to be largely deleterious, we exploited the principle that SAVs will be largely absent from polymorphisms common in the human population. Phase II HapMap SNPs represent both a high quality and extensive genome-wide set of human polymorphisms, as they have been genotyped for 270 individuals in four populations [27]. From this set of 3.1 million SNPs, we took all SNPs that fell in internal (that is, spliced) coding exons that were polymorphic in at least one individual and filtered them in the same way as SAVs (see Materials and methods). In addition, we only retained SNPs where we could determine the ancestral and derived allele with high confidence by utilizing orthologous positions in the chimp and macaque genomes. This approach allowed us to make an assumption of the allele directionality, which was important for detecting loss or gain of splice regulatory elements. The resulting dataset contained 15,547 SNPs with roughly equal numbers of synonymous and missense alleles (7,922 and 7,625, respectively). These SNPs fell within 13,163 individual exons from 7,038 genes. For ease of reference, we refer to this set of HapMap SNPs as 'hSNPs'.

Using these sets of variants, we carried out comparative analyses to identify the features that discriminate SAVs from hSNPs, which can be described at the sequence level (such as changes in the underlying splicing regulatory sequences and physical location within exons), or at the exon level (to predispose an exon to exon-skipping events) to enable a predictive framework for uncharacterized variants.

### Variant-based features

#### Changes in exonic splicing regulatory sequences

Our systematic assessment of all seven currently available ESR datasets examined their ability to identify splice-regulatory elements in the verified SAV sequences. This approach assessed the types of motif-altering changes associated with SAVs and provided benchmarking of the seven ESR collections (Table 1) to determine which most strongly differentiated real splice-affecting variants from common polymorphisms. Of these seven sets, two contain ESEs (ESEFinder and RESCUE-ESE), one represents ESSs (Fas-ESS) and the remaining four sets contain both ESEs and ESSs (PESX, NI-ESR, Ast-ESR and Composite-ESR). For both SAV and hSNP sequences we measured three possible types of changes in the ancestral versus derived allele (or wild type versus disease allele) as a result of the variant: ESR loss, ESR gain and ESR alteration (see Materials and methods).

**Figure 1:**
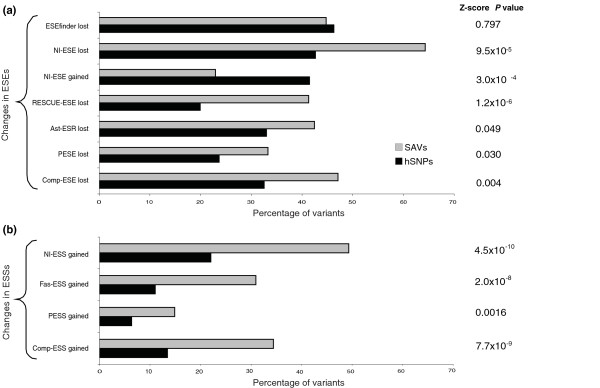
**Proportion of variants with gains or losses in exonic splicing regulatory sequence with significant differences between splice-affecting genome variants and HapMap SNPs**. SAVs were characterized by **(a) **the loss of ESEs and **(b) **the gain of ESSs. As a comparison, ESEfinder, Ast-ESR and PESE losses are also included. These were not significantly different between SAVs and hSNPs. Z score *P*-values from random bootstrap sampling relating to each type of change are located on the right of the histogram.

We first examined whether the proportion of SAVs with a particular type of ESR change was significantly different from that of hSNPs. Our comparative analyses identified two significant changes associated with variants that cause exon skipping: the gain of sequences defined as ESSs and the loss of sequences defined as ESEs (Figure 1; Additional file 3). Of these, we found that ESS gains had stronger discriminatory power than ESE losses. All the ESS datasets identified a significantly greater proportion of SAVs causing gains of ESSs. In contrast, results for ESE losses were split. NI-ESE, RESCUE-ESE and Comp-ESE showed a moderate but significantly greater proportion of ESE losses in SAVs than hSNPs. Losses of ESEfinder motifs were roughly equal between SAVs and hSNPs, both as a group of motifs and individually (Figure 1; Additional file 3). Nevertheless, we hypothesized that because the threshold set for each ESEFinder binding site is somewhat arbitrary, single base changes that cause a binding site to be 'lost' may not be functionally equivalent and that changes in certain positions may be less tolerated than others. We found one position in each binding matrix that occurred at significantly higher numbers in SAVs compared to hSNPs (by χ^2 ^test, *P *< 0.05; Additional file 4). Hence, there may be different functional constraints acting along the binding sites that are not properly captured by the default scoring thresholds and the position weight matrix scores as currently employed. Ast-ESRs, while not explicitly defined as ESEs or ESSs, showed no significant difference between variant groups for losses, alterations or gains [16]. Consistent with the direction of the previous ESR changes, SAVs were also significantly diminished for gains of ESEs using the NI-ESE dataset (Figure 1; Additional file 3).

#### The extent of ESR changes further differentiates SAVs from hSNPs

We investigated whether SAVs are further distinguished by the cumulative extent of the ESE losses and ESS gains. Many of the sets of putative ESRs are represented as hexamers (for example, RESCUE-ESE, NI-ESRs, PESXs, and so on), either because this is often the size of a single protein-binding site (for example, the GAAGAA ESE [28]), or because they are a reduced representation of larger binding sites. Because point variants may modulate several overlapping binding sites simultaneously, those affecting larger numbers of predicted sites are more likely to have significant impact, for which we assessed predictive power. The results showed that in all ESR sets except ESEfinder, numbers of ESS gains and ESE losses were much greater in SAVs than hSNPs (Additional file 3). We saw the greatest separation from hSNPs using NI-ESSs gains (98 gains in SAVs versus a mean of 32 in hSNPs, Z-score *P *= 1.92 × 10^-17^) and NI-ESEs losses (138 losses in SAVs versus a mean of 69 in hSNPs, Z-score *P *= 2.68 × 10^-10^), although RESCUE-ESE, Fas-ESS and Composite-ESR also give good, strongly statistically significant separations, despite the much smaller size of these datasets compared to NI-ESRs (Table 1).

**Figure 2:**
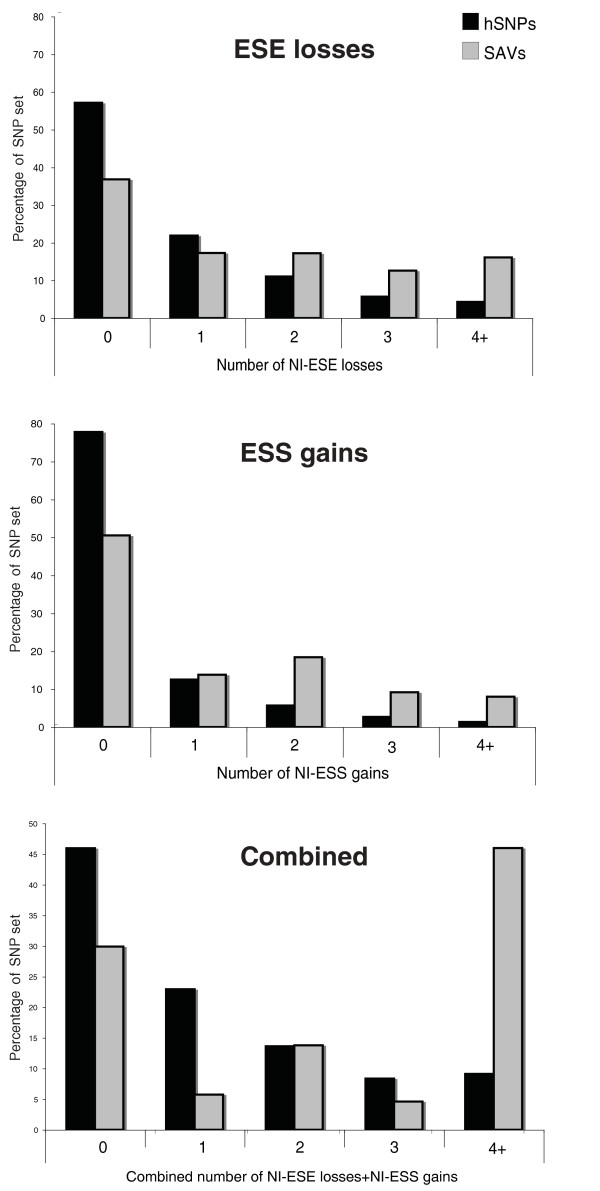
**Splice-affecting genome variants are characterized by losses of large numbers of NI-ESEs and the gain of large numbers of NI-ESSs, often in combination**. For both ESE losses and ESS gains, the proportion of SAVs with changes of two or more were significantly greater compared to hSNPs. Combinations of ESE losses and ESS gains, as opposed to each occurring independently, are highly enriched in SAVs compared to hSNPs (bottom graph).

For NI-ESR, losses or gains of two or more motifs were prevalent, with the divergence between SAVs and hSNPs becoming larger as the total number of occurrences increased (Figure 2a, b). When the extent of ESS gains and ESE losses were combined as a total number of changes, 46% of SAVs had four or more such changes compared to only 9% for hSNPs (Figure 2c). Furthermore, we compared the set of 80 experimentally verified splice-neutral variants against the hSNP dataset and found that no category of ESR change was significantly different (Additional file 3). This supports our assumption that hSNPs act as an appropriate proxy for splice-neutral variants and confirms that significant ESR differences are detectable between splice-affecting and splicing-neutral datasets.

Finally, using a recently established computational method [22], we investigated whether taking local RNA secondary structure into consideration improved the ability to distinguish functionally relevant ESR changes in SAVs from those in hSNPs. We found little evidence that local RNA secondary structure, as implemented by this method, improved our ability to differentiate these two datasets further (see Additional file 5 for methods and results).

#### Splice-altering sequence changes are under negative selection in common SNPs

**Figure 3:**
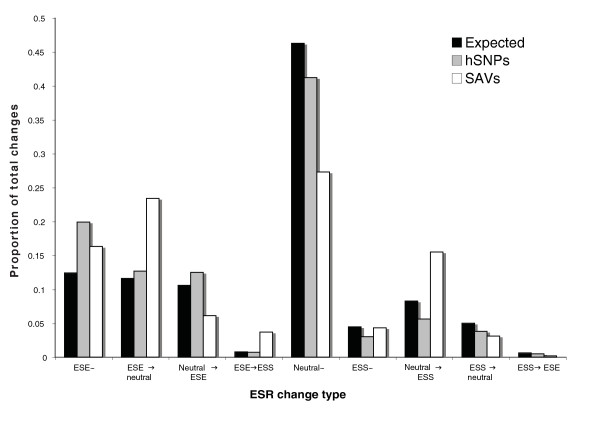
**Distribution of specific types of NI-ESR changes for SAVs and hSNPs compared to neutral expectation**. The tilde symbol (~) signifies an alteration where the hexamer is designated an ESE, neutral or ESS in both the wild-type and variant sequences. The arrow represents the direction of the change as a consequence of the change between wild type and variant hexamer. The neutral expected distribution reflects the underlying probability of each type of change given the ESE/ESS distribution among NI hexamers and the genome-wide nucleotide substitution bias in coding regions.

In the previous comparative analyses, we assumed that the differential signal in ESR changes between SAVs and hSNPs was a composite consequence of both functional ESR changes in SAVs and selective pressure to avoid those changes in common hSNPs [25]. To test this assumption, we investigated whether the proportion of each type of ESR change in SAVs and hSNPs, using the NI-ESR dataset, would differ when compared to an 'expected' neutral distribution created through permutation (see Materials and methods). This permuted distribution represents what we would expect if variants occurred randomly under no selective pressure for splicing. We found that while hSNPs followed the expected distribution closely for many of the changes, SAVs had almost two-fold higher proportions of ESS gains and ESE losses (Figure 3), confirming that these types of changes were a non-random, characteristic property of SAVs. Moreover, the highly significant difference in ESS gains between SAVs and hSNPs can be further explained by a significant reduction for this type of change in hSNPs compared to the expected distribution (5.6% of changes in hSNPs versus 8.3% under neutrality, χ^2 ^test *P *= 1.7 × 10^-8^), suggesting negative selection against the gain of silencers in common variants. We also identified a five-fold increase in the proportion of variants that cause direct changes from an ESE to an ESS in SAVs compared to both the expected and hSNP distributions (4.1% of changes in SAVs versus 0.8% under neutrality/hSNPs, χ^2 ^test *P *= 3.8 × 10^-12^; Figure 3), indicating that this type of change represents a strong indicator of splice-affecting changes.

#### Significant ESR changes in variants that increase exon inclusion

We carried out the same comparative analysis against hSNPs using a smaller set of 20 exonic variants that have been experimentally verified to cause increased exon inclusion (Additional file 6). Although lacking some of the statistical power of the larger exon skipping SAV set, we found that these variants were significantly enriched for ESSs losses (21 losses versus a mean of 5 in hSNPs, empirical *P *= 1 × 10^-4^; Additional file 3). They also exhibited greater numbers of ESE gains (25 gains versus a mean of 15 in hSNPs, empirical *P *= 0.034) and lower numbers of ESE losses (5 losses versus a mean of 16 in hSNPs, empirical *P *= 0.0097). These changes were the opposite of the changes caused by skipping SAVs and consistent with regulatory changes expected to increase exon definition. These results highlighted the antagonistic interplay between ESEs and ESSs in stabilizing or destabilizing exonic splicing.

#### Proximity to exon boundaries

**Figure 4:**
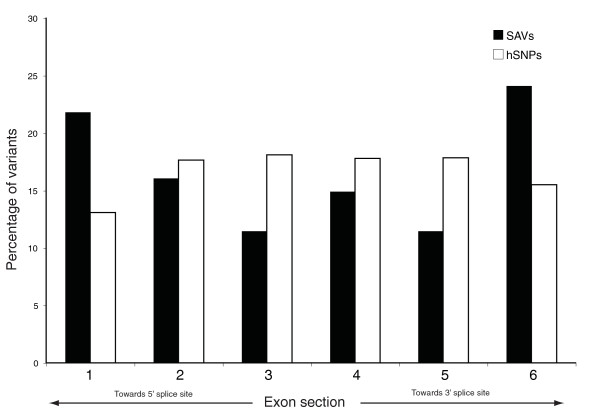
**SAVs are enriched at the borders of exons**. SAV and hSNP containing exons were divided into six equal sections and the proportion of variants falling into each section was plotted. While hSNPs were roughly distributed equally across the exon (with some depletion towards the edges), SAVs are significantly enriched at both edges of the exon (*P *= 0.005).

Previous studies have shown that a number of exonic characteristics are affected by proximity to the exon junction, including ESE density [25], evolutionary constraint [16,29] and codon bias [30]. Although circumstantial, this evidence supports the view that the boundaries of exons contain regulatory 'hotspots' that may be more critical to splicing than centralized regions. To investigate whether SAVs are more likely to be disruptive if located preferentially in these hotspot regions, we divided all SAV exons and HapMap exons into six equal parts and binned the SAV or hSNP variants according to their locations. Figure 4 shows that hSNPs were distributed roughly equally across the exons, with a small depletion at exon boundaries, whereas SAVs were enriched close to the exon boundaries and depleted towards the center (46% of SAVs located at the peripheral sections of exons versus 28.5% of hSNPs, *P *= 0.005). Nevertheless, over a quarter of the SAVs are located within the central sections of the exon, suggesting that while variants located at the peripheries of the exon are likely to have the greatest effect on splicing, other elements important for splicing may be found at positions across the exon, but not with discriminatory power for this analysis.

#### Regulatory evolutionary constraint of SAV regions

The availability of multiple sequenced mammalian genomes provides the opportunity for evolutionary comparisons of functional constraint across related species. Splicing patterns and exonic splicing regulatory elements are generally conserved across mammals [31]. Therefore, sequences important for splicing should be detectable by greater evolutionary sequence conservation; a case that is proven for intronic factors [32]. We hypothesized that the regions surrounding SAVs should be under greater evolutionary constraint than regions surrounding neutral variants. However, within coding exons, the constraint on the sequence due to splicing has to be decoupled from pre-existing protein-coding constraint. One solution is to measure conservation at synonymous codon positions, which are normally considered to be neutrally evolving. Several studies have demonstrated that ESRs increase selective constraint on synonymous positions [16,33]. An extreme example is the ultra-conservation of coding sequences that are associated with auto-regulatory alternative splicing of 'poison exons' in SR proteins [34].

**Figure 5:**
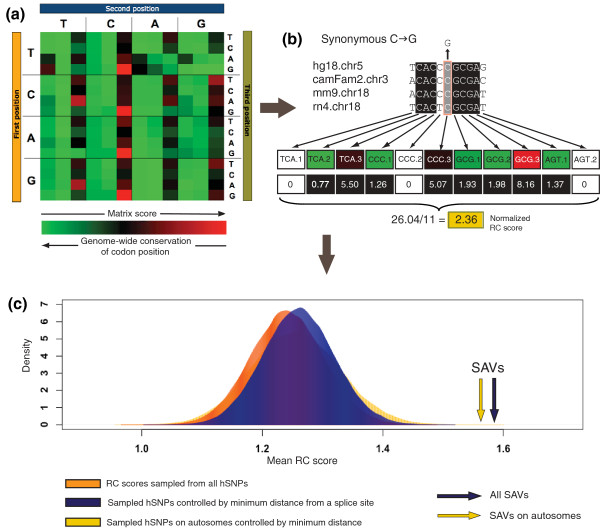
**Regions surrounding SAVs are under greater non-coding evolutionary constraint**. **(a) **We created a 192-codon position-specific scoring matrix based on genome-wide conservation levels across mammals. Matrix scores are visualized increasing from green to red. As scores are inversely proportional to the genome-wide conservation of each codon position, conservation levels can also be visualized using the same matrix, decreasing from green to red. **(b) **For each variant, four-way mammalian multiple DNA alignments were extracted for a region surrounding the variant, and a score assigned to each fully conserved column via the scoring matrix, and the total normalized by the length of the alignment. An example of a random synonymous CγG variant is shown. **(c) **The mean conservation score for all SAVs (blue arrow) and SAVs on autosomes (yellow arrow) was compared to a distribution of randomly sampled sets of scores from all hSNPs (orange distribution). Randomly sampled distributions of hSNPs were also created controlling for minimum distance from a splice junction by having similar distributions in this regard as SAVs (blue distribution). A distribution of mean conservation scores was also produced for hSNPs from autosomes also controlled by minimum distance from the splice site (yellow distribution).

To score regulatory constraint in coding regions, we created an expectation-based scoring matrix for each of the 192 positions of the genetic code. The scores were inversely proportional to conservation levels in genome-wide human/mouse/rat/dog DNA multiple alignments (see Materials and methods). By using a scoring scheme based on real evolutionary data, the scoring matrix not only preferentially scores synonymous over non-synonymous positions, but also incorporates other influences, such as codon bias and hypermutability. For example, the highest scores in the matrix are at synonymous positions in hypermutable CpGs (that is, TCG, ACG, CCG and GCG) as these are the least conserved coding positions genome-wide (Figure 5a). Using this scoring matrix, we calculated regulatory constraint (RC) scores in localized coding regions, representing all possible hexamer positions surrounding a variant, for all SAVs and hSNPs (Figure 5b) and compared the mean RC scores of all non-overlapping regions for each set. Results showed that sequences containing SAVs had significantly higher mean conservation scores than a random sampled distribution of hSNPs (1.583 versus a mean of 1.233 in hSNPs, Z score *P *= 5.71 × 10^-9^; Figure 5c, orange distribution).

We addressed a variety of sources of bias that could confound the outcome of the conservation analysis. For example, rates of synonymous and non-synonymous substitutions decrease close to splice junctions [29,30]. Data from hSNPs confirmed this result by showing that the RC scores were negatively correlated with distance from the splice junction (Additional file 7). However, since SAVs are enriched close to splice junctions, we repeated the analysis choosing hSNPs with similar distances from the splice junction as those in the SAV set. This shifted the hSNP distribution to greater mean RC scores (Figure 5c, blue distribution), but the difference with SAVs remained highly significant (1.583 versus a mean of 1.266 in hSNPs, Z score *P *= 1.92 × 10^-8^).

A second potentially significant source of bias was due to SAVs on the X chromosome contributing 35% of the variant set, compared to just 1.38% of the hSNP set. Prior SNP analyses identified the X chromosome as having lower rates of heterozygosity than autosomes [27], and human-mouse comparisons showed that genes on this chromosome were under greater evolutionary selection [35]. It was possible, therefore, that the prevalence of SAVs from the X chromosome contributed to the significantly higher conservation scores. We found that mean RC scores for hSNPs on the X chromosome were significantly higher than for other chromosomes (1.34 versus 1.24, Kolmogorov-Smirnov (K-S) test *P *= 0.008). Similarly, SAVs on the X chromosome had a higher mean RC score than SAVs on other chromosomes but the difference was not statistically significant (1.57 versus 1.67, K-S test *P *= 0.33) due to small sample sizes. We therefore repeated the analysis using only SAVs and hSNPs on autosomes (also controlling for distance from the splice junction; Figure 5c, yellow distribution). The difference in mean RC scores was further decreased but nevertheless remained highly significant (1.55 versus 1.25, Z score *P *= 1.28 × 10^-5^). Therefore, the predominance of SAVs from the X chromosome was not sufficient to explain the greater regulatory constraint surrounding SAVs.

We also examined whether SAV exons were more highly conserved than HapMap exons. We compared percent-identity from four-way multiple alignments, across entire exons or within non-synonymous positions of exons, excluding the X chromosome. No significant differences were found in mean percent-identities in non-synonymous positions (89% in SAV exons versus 88.7% in hSNP exons, Z score *P *= 0.122) or overall (77% in SAV exons versus 75% in hSNP exons, Z score *P *= 0.063). Furthermore, similar results were obtained using HapMap exons of all sizes, or those that closely resembled the size distribution of SAV exons. By controlling for alternative sources of constraint we concluded SAVs occur in regions of exons that are under greater non-coding constraint, indicative of negative selection for important function.

#### Exonic environment

We addressed features associated with exon definition to test whether exons containing SAVs (which we will term 'SAV exons') are significantly different in these aspects from exons containing hSNPs (termed 'HapMap exons') or from exons in general, indicative of a pre-existing weakness or predisposition to the effects of SAVs.

#### Exon size

A comparison of exon lengths between SAV and HapMap exons showed that SAV exons were significantly smaller (mean = 125.1 bp versus 197.8 bp, K-S test *P *= 1.269 × 10^-7^). However, further comparison of the SAV exons to internal exons from the Hollywood exon annotation database [36] showed that both the mean (125 bp versus 136 bp, *P *= 0.39) and median (112 bp versus 120 bp, *P *= 0.051) values of the SAV exons, although lower, were not statistically different in a randomized bootstrap analysis (see Materials and methods). When compared directly to constitutive Hollywood exons, HapMap exons were significantly larger (K-S test *P *< 2.2 × 10^-16^). We examined the potentially confounding problem of larger HapMap exons through simulation analyses and showed that the probability of an exon containing a SNP increased as exon length increased (see Materials and methods). The simulated exons with SNPs had the same length distribution as HapMap exons (Additional file 8). We therefore controlled for equivalent exon size in all subsequent analyses.

#### Splice site strengths

Signals critical for exon definition are the 5' and 3' splice sites and branch point. The strength of these signals may influence whether an exon is constitutively or alternatively spliced, creating conditional dependency on ESEs and vulnerability to their loss. We found that the mean 5' and 3' splice site scores were lower in SAV exons than HapMap exons but were not statistically significant (Table 2). Assessing exons with large numbers (≥ 2) of NI-ESEs losses and/or NI-ESS gains revealed stronger 3' splice site scores in HapMap exons than SAV exons (Table 2), suggesting stronger 3' splice sites may shield some HapMap exons from the effects of ESR-changing SNPs. Nevertheless, the large overlap in splice site strengths between these two groups indicated that splice site strength could not be used to uniquely predict SAV vulnerability in exons.

**Table 2:** Significance of exon and intron-related features for skipping SAV and HapMap exons

Exon feature	SAV mean	hSNP sampled mean	Z-score	*P*-value
Exon splice junction strength				
Exon 3' SS score (all exons)	7.811	8.489	-1.90	0.057
Exon 5' SS score (all exons)	7.885	8.302	-1.43	0.154
Exon 3' SS score (with ESR)	7.568	8.534	-2.03	0.022
Exon 5' SS score (with ESR)	8.008	8.371	-1.71	0.230
				
Exon ESR density				
ESEfinder density FL	0.126	0.152	-3.78	1.53 × 10^-4^
NI-ESE density FL	0.323	0.372	-3.37	7.37 × 10^-4^
NI-ESS density FL	0.133	0.093	4.30	1.67 × 10^-5^
ESEfinder density W40	0.129	0.153	-3.15	0.0016
NI-ESE density W40	0.324	0.379	-3.47	5.18 × 10^-4^
NI-ESS density W40	0.140	0.094	4.50	6.85 × 10^-6^
				
Intronic ESR densities				
Upstream NI-ESE density	0.201	0.224	-1.55	0.122
Downstream NI-ESE density	0.235	0.250	-1.06	0.314
Upstream NI-ESS density	0.295	0.241	2.44	0.014
Downstream NI-ESS density	0.258	0.210	2.36	0.018

For each feature, the mean values for non-redundant SAV exons were compared to a bootstrap distribution of sampled means for HapMap exons of similar sizes (hSNP sampled mean). For exon splice junction strength, results marked 'all exons' indicate that the comparison was done using all exons in both datasets and those marked 'with ESR' indicate comparisons using only exons containing a variant with splice-associated ESR changes, that is, ESE loss and/or ESS gain. For exon ESR densities, densities were either measured across the full length of the exon (FL) or in windows of 40 bp at either side of the exon (W40). For exons <80 bp in length, the W40 density is the same as full length density to avoid redundancy. Intronic ESR densities were measured in the first 100 bp upstream and downstream of the exon. SS, splice site.

#### ESR density in exons and introns

**Figure 6:**
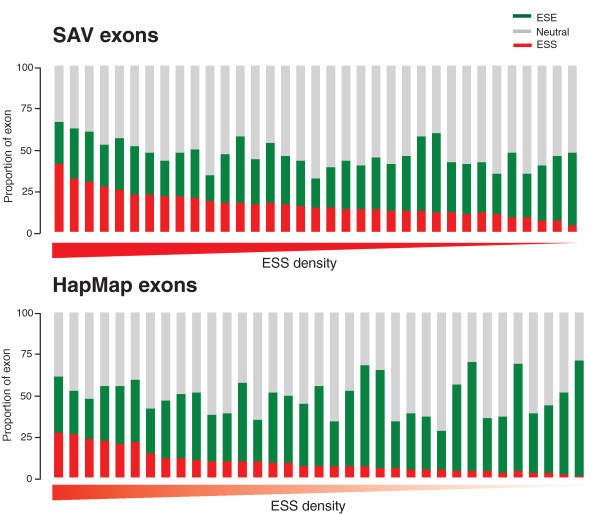
**Exons containing SAVs have significantly lower ESE and significantly higher ESS densities than exons containing hSNPs**. As an illustration, the proportion of overlapping hexamers that are considered ESEs (green), ESSs (red) or splice neutral (grey) was plotted for 35 exons containing SAVs (that cause ESE/ESS changes) and a set of 35 randomly selected, length-matched hSNP-containing exons. Exons in both sets are sorted in descending order by ESS density.

A major feature postulated to distinguish exons from introns is higher densities of ESEs and low or absent densities of ESSs. The exact opposite is true of introns and pseudoexons. We therefore looked at the density of exonic splicing regulators in SAV and HapMap exons using the NI-ESRs. We found that SAV exons have significantly lower densities of ESEs and higher densities of ESSs across the exon length (Table 2 and Figure 6). To confirm that these were features specific to SAV exons rather than something particular to HapMap exons, we repeated the comparison to random genome-wide exons and found very similar results, suggesting that this is a feature characteristic of SAV exons. ESR densities of SAV exons are, in many cases, more comparable to an intronic environment represented in flanking introns (mean ESE density = 0.26, mean ESS density = 0.2; see Materials and methods). Moreover, directly flanking SAV exons, we found that intronic sequences showed higher densities of ESSs and slightly lower densities of ESEs than around hSNP exons (Table 2).

#### Variants that activate *de novo* ectopic splice sites

**Figure 7:**
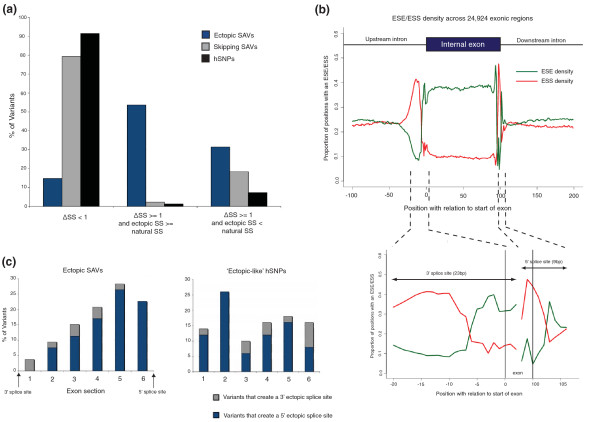
**Features that characterize variants that activate *de novo *ectopic splice sites ('ectopic SAVs')**. **(a) **Most ectopic SAVs, in contrast to hSNPs and skipping SAVs, have a large Δ*SS *value and create an ectopic splice site that is stronger than the natural splice site. **(b) **Hexamers in the vicinity of the splice junctions are largely made up of ESSs. The graph represents the proportion of positions occupied either by an ESE or ESS motif across approximately 25,000 internal exons. Each position on the graph represents the first base of a hexamer sliding across 100 bp of the upstream and downstream introns and the first and last 50 bp of the exon. **(c) **Ectopic SAVs are located predominantly in the vicinity of the splice site of the same type created, that is, the majority of ectopic splice sites created are 5' ectopic sites and are located towards the end of the exon close to the 5' splice site. hSNPs that create a strong ectopic splice site computationally ('ectopic-like' hSNPs) are distributed across the exon in quite the opposite way, indicating the same constraints do not apply to these variants.

Next, we assessed features that define exonic variants that create *de novo *ectopic splice sites. We used a set of 54 experimentally verified examples of *de novo *ectopic splice site variants (Additional file 9) to discern features that distinguish our two sets of SAVs (that is, 'ectopic SAVs' and 'skipping SAVs') from each other and from hSNPs. First, to measure splice site creation, we used a simple metric, Δ*SS*, to measure the maximum difference in splice site scores between these two sequences for all possible 5' and 3' splice sites around the variants (see Materials and methods). A large positive delta score suggests a change in the surrounding sequence towards a better scoring splice site. Requiring a relatively low Δ*SS *score of at least 1 captured the majority of ectopic SAVs (approximately 85%) compared to 20% of skipping SAVs and 8% of hSNPs. We also compared the highest-scoring variant-generated splice site to the natural splice site score. Over half of ectopic SAVs created ectopic splice sites that were comparable to or stronger than the natural splice site, in contrast to a tiny proportion of skipping SAVs and hSNPs (Figure 7a). Thus, these two metrics represent excellent features to discriminate ectopic SAVs from splicing SAVs. In support of this conclusion, one of the two exon skipping SAVs we predicted to also create strong ectopic splice sites, a synonymous mutation in the *ATR *gene, has been shown experimentally to cause a combination of both exon skipping and ectopic 5' splice site activation [37].

An additional feature of ectopic SAVs was a highly significant excess of ESS gains (*P *= 2.85 × 10^-15^) and ESS alterations (*P *= 1.95 × 10^-3^) compared to hSNPs, similar to that seen in skipping SAVs. The degree of ESS gains in ectopic SAVs was even greater than that for skipping SAVs, averaging 1.28 ESS gains per variant compared to 1.12 for skipping SAVs and 0.39 in hSNPs. When averaged across all internal constitutive exons, we found NI-ESS density spiked near splice junctions (Figure 7b), which was consistent with previous studies on smaller ESS datasets [15,38], suggesting a possible explanation for the excess in ESS gains. To address this further, we compared the ectopic SAVs to a set of 54 hSNPs that were tightly scored as 'ectopic-like' (but showed no evidence of splice site creation in mRNA or EST datasets; see Materials and methods). We found that ectopic SAVs had almost a 2.5-fold greater number of ESS gains (68 versus 28) and a 1.8-fold greater number of ESS alterations (23 versus 13), despite both sets having similar distributions of maximum ectopic splice site scores (KS-test *P *= 0.11). The process of creating strongly scoring splice-site consensus sequences could not, therefore, fully explain the enrichment in ESS changes in SAVs. Additional ESS creation may facilitate activation of the new ectopic splice sites by inhibiting the natural splice site. This is consistent with a functional study by Wang *et al. *[38] whereby ESS motifs placed between competing 5' and 3' splice sites consistently inhibited the use of the intron-proximal splice site.

Finally, the location of ectopic SAVs and 'ectopic-like' hSNPs across exons revealed very different distributions (Figure 7c). Ectopic SAVs were predominantly located in the half of the exon closest to the natural splice site they replaced. The reverse was true of 'ectopic-like' hSNPs, which were distributed across the exon in an opposite manner. These differences, in addition to the lack of silencer gains, likely account for the lack of activity of these 'ectopic-like' hSNPs.

#### Skippy - a web tool for the detection of splice-modulating exonic variants

It is important for researchers screening for causative variants associated with disease to have access to user-friendly bioinformatics tools that can score variants for relevant splice-associated features. In this way, variants can be either prioritized for further splicing-based functional assays or the results can be used to further elucidate the mechanism of aberrant splicing when a causal variant has been implicated. To this end, we developed a publicly accessible web-based tool, Skippy, to allow users to rapidly score human exonic variants for all relevant exon-skipping features identified in this study. As well as these features, Skippy can also be used to identify potential ectopic SAVs.

Unlike other splicing assessment tools that require laborious extraction of the exonic/intronic sequence for input and only allow a single sequence to be submitted at a time (for example, [22,39]), Skippy requires only the chromosomal location and identity of the variant alleles as input, accepting up to 200 variants at a time. Results are returned in HTML tabular form as well as a tab-delimited text file. To facilitate interpretation of results, all scored features can be compared to distributions of hSNPs from similar genomic contexts. For example, the RC score for a candidate variant can be compared to a distribution of RC scores for hSNPs having similar features, such as equivalent minimum distances from the splice junctions. The web tool is freely available at [40].

## Discussion

The emergence in recent years of high throughput genotyping and resequencing technologies provides an unprecedented opportunity to identify disease alleles associated with both common and rare disorders. As functional characterization is highly laborious and time consuming, computational prioritization is a preferred approach to assessing disease candidates. Exonic mutations are traditionally assessed for an effect on protein function; however, those that are translationally silent are often overlooked for roles in exon skipping and ectopic splice site creation. Moreover, variants are traditionally only considered in the vicinity of splice sites if they fall directly at splice boundaries, whereas we have shown that SAVs are enriched in regions near, but not at, the splice junctions. Any of these seemingly innocuous sequence changes may have greater consequences for gene function than a single missense mutation. We therefore showed that SAVs have novel features distinguishing them from common human polymorphisms through a succession of bioinformatics approaches and built a novel web tool for examining genomic sequence changes that are likely to affect splicing.

### Exon skipping SAVs cause local changes in splice regulatory elements

Our comparative analyses identify two main types of ESR changes associated with exon skipping: the gain of sequences defined as ESSs and the loss of sequences defined as ESEs. We are the first to report that all ESS datasets showed a strong statistical enrichment for gain of ESSs in known SAVs with a moderate to high signal to noise ratio. Surprisingly, we also found that solely considering whether a variant causes ESE loss was a relatively weak predictor of splice-affecting events. Although the widely used ESEfinder matrices did not discriminate the known SAVs from the control groups, other ESE datasets (NI-ESE, RESCUE-ESE and Comp-ESE) showed statistically significant enrichments for ESE loss.

The study of ESR changes may not be a binary endeavor, as a single SNP can affect a number of putative overlapping binding sites. We found that SAVs are more strongly associated with the loss of large numbers of ESEs and the gain of large numbers of ESSs. This analysis also highlighted the neighborhood inference set of putative ESRs ('NI-ESRs') as providing the strongest signal for exon skipping variants. NI-ESRs are a relatively new set of predicted splice regulatory elements and have therefore been little used in clinically associated splicing studies to date. The neighborhood inference algorithm greatly enlarged the set of previously known ESEs and ESSs to cover over a third of all possible hexamers, increasing the likelihood of false positives in our ESR change analysis. We nevertheless saw impressive separation between SAVs and hSNPs, suggesting that many of these novel ESRs represent functional elements. None of our test set of known exon-skipping variants was originally identified, nor confirmed using this dataset.

As an illustration, a published missense mutation in exon 12 of the *HEXB *gene causes full exon skipping and is responsible for chronic Sandhoff's disease. The variant was identified experimentally and subsequently predicted to cause the loss of two ESEfinder sites [41]. Our analysis using NI-ESRs revealed that this mutation caused the loss of five overlapping ESEs and the creation of two overlapping ESSs (both of which were direct conversions from ESEs to ESSs). Notably, this extent of NI-ESR changes, unlike those for ESEfinder, scored as highly discriminative for SAVs compared to hSNPs. Furthermore, we found that concurrent loss and gain events were better predictors than single events. This fact is illustrated by the synonymous skipping mutation of exon 7 in *SMN2 *that destroys two overlapping ESE hexamers and creates two overlapping ESS hexamers. Functional studies of SF2/ASF and hnRNPA1 binding in this exon proved that reduced binding of SF2/ASF [11] and increased binding of hnRNPA1 [42] were responsible for reduced inclusion of the *SMN2 *exon.

### Increased silencer activity is likely for many SAVs

Although the loss of ESEs is the most commonly assigned change in published splice-associated variant studies, increased silencer function was seen in 37% of our known exon skipping SAVs, in which each caused the gain of two or more ESSs. The clear enrichment for silencer creation in SAVs and selection against silencer acquisition in common polymorphisms suggests that this may be a major mechanism responsible for exon skipping. Furthermore, for mutations in which the mechanism of action has been experimentally studied, with the exception of *SMN2*, none were studied for the possibility of increased silencer function. The importance of exonic silencers in splicing is further highlighted by our results showing that SAVs that cause increased exon-inclusion are likely to operate largely by the loss of ESSs. We conclude that newly created ESS sites also facilitate formation of *de novo *ectopic splice sites. The action of inhibiting a natural existing proximal splice site, as ESS are known to do, would be similar to those causing exon skipping when no other alternative splice site was available.

### Caveats

It is important to note that despite the strong signals we identified, there are a number of limitations to solely using ESR analyses in a predictive manner. For example, even using the NI-ESR set, some SAVs were not captured with expected regulatory changes. Around 9% of the SAVs had no relevant changes in any of the ESR datasets, indicating that putative ESRs do not cover the full spectrum of functional splicing regulatory elements or these variants act through an alternative mechanism (for example, RNA secondary structure). Furthermore, context is very important for ESR function. This fact was highlighted by a recent study of 'designer' exons that placed different combinations of known ESEs and ESSs within a minigene exon and found that exons with the same proportion of enhancers and silencers exhibited highly variable inclusion levels that were context specific according to the order of regulatory elements across the exon [43].

### Exon skipping SAVs occur in weakly defined exons

Our analyses of the exonic environment suggested that an exon-skipping outcome was not necessarily solely dependant on the changes in splice regulatory elements, but may also be influenced by pre-existing features of exon definition. In this analysis SAV exons were not discernibly weaker at splice sites than other exons. However, experimental studies have indicated that weak splice sites are a factor. For example, the 5' splice site of *SMN2 *exon 7 was reported to be suboptimal through experimental and compensatory analyses [44]. This finding was not reproducible using solely computational scoring, highlighting the limitations of current *in silico *methods in detecting subtle but potentially significant features of exon definition.

Along with context and strength of the splice sites, exon definition is influenced by ESE and ESS motif densities [43]. It is revealing, therefore, that SAV exons have significantly lower densities of ESEs and significantly greater densities of ESSs - a clear attribute of weak exon definition. It is currently thought that splicing efficiency increases linearly as the number of enhancer elements increases because the role of multisite splice-regulatory elements is to increase the probability of an interaction between the regulatory complex and the splicing machinery [45,46]. Conversely, as the number of silencer elements increases, splicing efficiency decreases [43]. Indeed, we found that ESS density of many SAV exons was more comparable to that of introns than exons. As weakly defined exons, they appear vulnerable to variants that further modulate the ESE/ESS density. Illustrating the point, some exons are vulnerable to exon skipping by numerous SAVs. Seven SAVs occur in constitutively spliced exon 12 of the *CFTR *gene [47,48], which has a low ESE density (0.280 versus 0.371) and exceptionally high ESS density (0.293 versus 0.091) compared to mean densities in HapMap exons.

Our results also suggest that ESS elements in the introns may play a role in the susceptibility of exons to SAVs. However, the function of ESSs in introns is not fully elucidated [49,50]. If ESSs in introns act mainly as intronic splicing silencers, they may make the exon increasingly reliant on exonic splicing enhancers. Such a case has been demonstrated for one of the SAVs in exon 7 of *SMN1*/*SMN2*, where removal of a flanking intronic splicing silencer sequence compensated for the exon skipping effect [51].

## Conclusions

It is becoming increasingly clear that both missense and synonymous mutations within exons can have devastating effects on gene function by modulating splicing. The location of these mutations in coding sequence, as well as the lack of a clear strategy for their identification, means that their effects are often overlooked. As a consequence, known examples are currently small in number, but are likely to be underestimated. This work provides the first large-scale analysis of exon skipping variants to computationally characterize their genomic context. We identified a number of features associated with the variants and their exonic and intronic environments that are significantly different from common splicing-neutral polymorphisms. Exon skipping SAVs are characterized by extensive loss of exonic splicing enhancers and gain of splicing silencers, often in combination. They tend to occur in regions close to splice sites and in regions under greater non-coding evolutionary selection. They also tend to occur in exons with a fairly weak environment for exon definition that is the likely cause of their vulnerability to skipping events.

Our comparative approach proved robust in identifying relevant features in other types of SAVs too. Variants that cause increased exon inclusion are characterized by ESS loss and, to a lesser degree, the gain of ESEs. Variants that activate an ectopic splice site simultaneously create large numbers of ESSs, in addition to a strong consensus splice site, and inhibit use of the natural splice site. These results provide greater insights into the possible mechanism of action of these variants and should improve strategies for identifying disease candidates. To this end, we have developed a web-based tool, Skippy, to score candidate human genomic variants for features predictive of an exon-skipping outcome or creation of an ectopic splice site.

## Materials and methods

### Collation of a set of known exonic variants causing exon skipping

In total we collated a set of 87 SAVs by extracting synonymous and missense variants from the Alternative Splicing Mutation Database [26] (with a splicing effect score <0), and from our own extensive literature searches. Only single-point variants that had been experimentally verified for exon skipping were used in the reference set. We excluded the following: nonsense variants [3] (that is, those that create a stop codon); and variants that affect the splice junction (that is, 3 bp or less from either splice junction). Genomic positions for all 87 identified cases (32 synonymous, 55 missense) were mapped back onto the reference human genome (assembly Hg18). For the analysis of the types of ESR changes involved in increased exon inclusion, we used a set of 20 variants from the Alternative Splicing Mutation Database with splicing effect scores >0 (7 synonymous, 13 missense).

### Obtaining a comparator set of putatively splicing-neutral coding SNPs

All 'phase II' HapMap SNPs (release 22), termed 'hSNPs', that were polymorphic in at least one individual were downloaded from the website [27]. SNPs had to fall within an internal coding exon (using the Ensembl known gene set, v45.36 g) and more than 3 bp away from a splice junction. Directionality of mutations (that is, the derived alleles) utilized three-way human-chimp-macaque MulitZ alignments (hg18, panTro2, rheMac2) obtained from the UCSC Genome Browser [52] via Galaxy [53]. SNPs were retained only if there was a full three-way alignment available, chimp and macaque bases were identical, and one of the human alleles matched the ancestral chimp-macaque base. hSNPs included within the set of known SAVs were excluded from the comparator set (rs2306159, rs4647603 and rs2295682 [54], rs688 [55] and rs17612648 [56]). In addition, four hSNPs (rs17658212, rs4963793, rs591 and rs3818562) with reported correlations to splicing changes (but unverified) [57] were also excluded. A total of 15,547 hSNPs (7,922 synonymous, 7,625 missense) were obtained. Derived allele frequencies of >5% and >10% in at least one population were assessed. We found no appreciable difference for any of our analyses when using SNPs with greater derived allele frequencies. In addition to our hSNP comparator set, we also identified a set of 80 variants from the literature that have been experimentally tested in mini-gene assays and found to have no effect on exon splicing (Additional file 2).

### Changes in exonic ESRs

For our analysis, we obtained six sets of ESR sequence prediction datasets. Three comprised sets of bioinformatically defined hexamers (RESCUE-ESEs (238 ESEs) [58], NI-ESRs (979 ESEs and 496 ESSs) [19] and Ast-ESRs (285 undefined hexamers) [16]). PESX has bioinformatically defined octamers (2,096 PESEs and 974 PESSs) [18]). Fas-Hex2 contains experimentally defined ESS hexamers (176 ESSs) [15]). ESEfinder has four experimentally defined position weight matrices for SR protein binding sites [59]. Composite-ESRs are a combined set of hexamers derived from PESX, RESCUE-ESE, and Fas-Hex2 ESS, representing 400 ESEs and 217 ESSs [60]. The effect of SNP changes on ESR predictions was calculated using a sliding window that covered all hexamers surrounding the variant. N-mers that did not 'score' as an ESE or ESS were considered splicing-neutral. Comparisons between the wild-type sequence (or ancestral allele) and the variant sequence (or derived allele) measured ESR loss (for example, an ESE to a neutral), ESR gain (for example, neutral to an ESE) and ESR alteration (for example, ESE to a different ESE). In the case of NI-ESRs, PESXs and composite ESRs, ESEs and ESSs were considered separately. For the analysis of changes in NI-ESRs, the types of changes between alleles were counted for all overlapping hexamers in which the variant was present. Expected proportions for each of the nine categories of change were calculated by permutating every base of 4,096 hexamers to all remaining bases (for example, A would be permutated to T, G and C) to give 73,728 (4096 × 3 × 6) permutations. Base substitution biases were taken into account by measuring base substitutions in the hSNP derived allele set (Additional file 10) and for each permutation, weighting the ESR-change category by the proportion of base substitutions of that type.

### Regulatory evolutionary constraint

An expectation-based scoring matrix measuring regulatory constraint in coding sequences was created by measuring the proportion of columns fully conserved for each of the 192 codon positions using a randomly selected set of 62,000 internal human exons in 6,428 genes from Ensembl (v47.36i). Exons were distributed genome-wide and had conserved counterparts in mouse, rat and dog genomes. Scores were assigned for each codon position by (1 - Pr_*CODi*_) × 10 where Pr_*CODi *_is the proportion of columns in all the alignments that were fully conserved for codon _COD_, position *i*. Scores for each codon position are therefore weighted so that they are inversely proportional to their overall conservation level. Conservation scores, measuring non-coding constraint in coding sequence, were calculated for regions surrounding variants in the hSNP and SAV sets. Orthologous sequences from human, mouse, rat and dog were extracted from 17-way MultiZ multiple alignments from the UCSC Genome Browser [52] for 5 bp either side of the SNP (representing all hexamers containing a SNP (a total of 11 bp) using Galaxy python scripts [53]. Smaller flanking regions were extracted if the variant was located less than 5 bp from the splice junction. Only ungapped alignments containing at least two species in addition to human were used. The RC score surrounding a variant RC_Var _was calculated as follows:

where *N *is the number of columns in the alignment, *i *is the column position, *S*_*i *_is the conservation status of the column (1 for conserved across the alignment, 0 if not fully conserved) and δ_*ci *_is the weight of the score depending on the codon position of the sequence of *i *in human (using the 192 codon scoring matrix). Pre-computed conservation scores for each base of all internal coding exons in the human genome (assembly Hg18) are available as a custom wiggle track on the UCSC genome browser from [40]. For all statistical analyses, only variants with non-overlapping regions were used to avoid bias. To compare conservation in SAV exons and HapMap exons, human/mouse/rat/dog multiple alignments were extracted across all exons represented in both sets. We computed the proportion of non-synonymous sites and proportion of columns that were fully conserved across the alignment within each exon.

### Exon-based features

All exons (and their flanking intronic sequences) containing SAVs and hSNPs were extracted from the human genome (assembly Hg18) using the Ensembl API [61] always using the largest exon isoform (except in the case of intron retention events). A genome-wide set of internal human cassette exons was downloaded from the Hollywood exon annotation database [36]. We retained exons between 20 and 1,000 bp with canonical GT-AG splice junctions, solely annotated as constitutive or alternatively spliced, obtaining 105,932 exons. Of these, 98,692 were annotated as constitutive and 7,240 were alternatively spliced. A simulated distribution of expected exon lengths for hSNPs, given a random distribution across the genome of 1 every thousand bases [27], was calculated for each exon length *n *(going from 20 bp to 1,000 bp) using the formula *fr*(*n*) = *pSNP *× *obs*(*n*) where *pSNP *= 0.001 and *obs*(*n*) is the observed number of exons for length *n *in the set of Hollywood exons. Splice site strength at both the 5' and 3' splice junctions was measured using the MaxEntScan maximum entropy scoring program [62] with default settings. We calculated ESR density within an exon by scanning a window of size *n *(depending on size of the ESR) across the length of the exon, and then dividing by the number of windows that scored as an ESE or ESS by the total number of windows. ESEfinder densities were calculated differently due to their encoding as position weight matrices of differing length. The density of each of the four position weight matrices within the exon was calculated separately using the windowing method and summed to make an overall density. We excluded the possibility of ascertainment bias for exon features due to expression levels by comparing 68 exons from SAV-containing genes that contained hSNPs but not SAVs to the rest of the hSNP exon dataset. We found no significant differences for ESR change or exon characteristics (such as exon length, splice site strength, ESE/ESS density, and so on) compared to other hSNPs or their exons.

### Intron-based features

All ESE/ESS densities of intronic sequences were measured using the NI-ESR set in the same way as for exons, on 100 bp of sequence directly flanking each side of the exon (excluding the conserved GT-AG splice site dinucleotides). Any exons with a flanking intron of less than 102 bp were excluded.

### Variants that activate *de novo* cryptic splice sites

From the DBASS3 [63] and DBASS5 [64] databases, we obtained 54 experimentally verified examples of exonic mutations that activate a *de novo *(that is, not pre-existing) ectopic 5' or 3' splice site and are located more than 3 bp away from either splice junction and mapped them back on to the human genome assembly hg18 (Additional file 9). We measured potential creation of *de novo *splice sites by a variant using a metric Δ*SS*. Δ*SS *represents the maximum change in values for either 5' or 3' MaxEnt splice site scores between variant and wild type, that is, Δ*SS *= *max*(Δ*5'SS*|Δ*3'SS*). Δ*5'SS *= (*ME*_*var *_- *ME*_*wt*_) where *ME*_*var *_and *ME*_*wt *_are the 5' MaxEntScan scores for the sequence including the variant and wild-type allele, respectively. Similarly Δ*3'SS *is calculated in the same way but using the 3' MaxEntScan scoring program. Δ*5'SS *and Δ*3'SS *were calculated for every appropriate sequence window (9 bp for 5' splice sites and 23 bp for 3' splice sites) in which a variant could play a role, sliding the window 1 bp each time. A comparator set of the top 54 ectopic-like hSNPs were created by choosing those hSNPs with the greatest scores for putative ectopic splice sites created by the variant, a Δ*SS *≥ 1 and no evidence of ectopic splice site creation as judged by mRNA and EST evidence from GenBank. Interestingly, prior to using the last filter, we found two of the top 56 hSNPs have strong evidence of causing ectopic splice site creation (rs7529443 (G->A) and rs2863095 (G->A)). This strategy, used with other evidence, can be used to identify novel ectopic splice site creating SNPs. To identify whether natural splice sites are predominantly made up from sequences defined as ESSs, we used DNA sequence from 100 bp within the exon (the first and last 50 bp in cases where the exon length >100 bp) in addition to 100 bp from the flanking upstream and downstream introns from a subset of the constitutively spliced exons with canonical GT-AG splice junctions from the Hollywood database. We therefore required that exons be at least 100 bp in length and contain flanking introns of at least 200 bp in length (so as not to contain mixed signal from nearby exons), leaving 24,924 exons.

### Statistical analysis

Unless otherwise indicated, we carried out a bootstrap analysis to compare SAVs against the hSNP set by randomly sampling sets of the same size and proportion of synonymous and non-synonymous as the SAVs without replacement (using the Perl module Math::Random) from the hSNPs 1 × 10^5 ^times. For the analysis involving ectopic SAVs, only hSNPs with a Δ*SS *score of 0 were compared. The number of cases sampled from the hSNP set for bootstrap analysis depended on whether the parameter was variant-based (that is, dependant on the variant, such as changes in ESRs) or exon-based. For variant-based parameters, all variants were used. As some SAVs or hSNPs fall within the same exon, exon-based parameters utilized only unique exons within the set to avoid biasing the analysis. Z-scores were calculated as long as the distribution of sampled values passed the Shapiro-Wilk test for normality (*P *> 0.05) otherwise the lowest empirical *P*-value was presented. *P*-values were derived from Z-scores calculated using:

where *x *is the feature value (or mean value) for SAVs, *μ* is the mean and *σ* is the standard deviation of a distribution of feature values (or mean values) of randomly sampled hSNPs. A more stringent α value of 0.01 was used to determine statistical significance given the large number of statistical comparisons carried out. Comparison of the proportion of SNPs showing changes in different motif positions within ESEfinder motifs for SAVs and hSNPs was carried out using χ^2 ^with Yates correction. Exon length distributions were compared using both the sampling approach above, as well as the K-S test as implemented in the R statistics package.

## Abbreviations

bp: base pair; ESE: exonic splicing enhancer; ESR: exonic splicing regulatory sequence; ESS: exonic splicing silencer; EST: expressed sequence tag; hnRNP: heterogeneous nuclear ribonucleoprotein; hSNP: HapMap single nucleotide polymorphism; K-S: Kolmogorov-Smirnov; NI: neighborhood inference; RC: regulatory constraint; SAV: splice-affecting genome variant; SNP: single nucleotide polymorphism; SR: serine/arginine rich.

## Authors' contributions

AW collated all splice variant datasets, designed and performed the experiments, analyzed the data, coded and implemented the Skippy program/webtool and drafted the manuscript. JCM contributed the hSNP variant dataset and conceived of the SNP density/exon length analysis. LE conceived of the study, and participated in its design and coordination and helped write the manuscript. All authors read and approved the final manuscript.

## Supplementary Material

Additional file 1 List of 87 synonymous and missense splice-affecting genome variants (SAVs) that cause exon skipping used for analysis in this study. The variants are derived from [12,13,37,41,44,47,48,54-56,65-103]. Click here for file

Additional file 2 List of 80 synonymous and missense variants that have been experimentally tested in mini-gene constructs and do not cause changes in splicing. The variants are derived from [74,104-106]. Click here for file

Additional file 3 (a) Full results of ESR changes and bootstrap analysis of exon skipping SAVs vs. hSNPs, (b) splice-neutral variants vs. hSNPs, (c) SAVs that cause exon inclusion vs. hSNPs, (d) ectopic SAVs vs. hSNPs and (e) ectopic-like hSNPs vs. hSNPs with a ΔSS of 0. Click here for file

Additional file 4 Set of graphs illustrating that exon-skipping SAVs are significantly overrepresented within certain positions across the four ESEfinder matrices. Click here for file

Additional file 5 Methods and results for an analysis on whether using local RNA secondary structure as a filter improves our ability to distinguish exon skipping SAVs from hSNPs. Our results suggest that using this filter does not improve our ability to predict SAVs although a small number of SAVs may arise from the indirect uncovering of ESS motifs by changes in local RNA secondary structure. Click here for file

Additional file 6 List of 20 variants that cause increased exon inclusion. The variants are derived from [44,47,104,107,108]. Click here for file

Additional file 7 Two plots that show that mean RC score is negatively correlated with minimum distance from a splice junction (top) but not correlated with exon length (bottom). Click here for file

Additional file 8 Only distributions of exon lengths up to 600 bp were plotted for clarity. Genome-wide exons were divided into constitutively spliced (CE) and alternatively spliced (AS) as defined by the Hollywood database [36]. A fifth, expected set of exons represents a set of exon lengths we would expect given the average distribution of hSNPs across the genome and fits the real distribution of HapMap exons closely. Click here for file

Additional file 9 List of 54 variants that cause *de novo *5' or 3' ectopic splice site activation. The variants are derived from [4,37,86,109-154]. Click here for file

Additional file 10 Base substitution distributions in hSNPs were used a background nucleotide substitution rates in calculating an expected distribution of ESE/ESS changes of the NI-ESR hexamer set (Figure 3). Significant differences in distributions between skipping SAVs and hSNPs (such as that seen in A->T, T->C and G->T) and ectopic SAVs (A->T, A->C, T->A, C->G) while potentially of biological interest, should be treated with caution due to small number of skipping and ectopic SAVs and large discrepancy in dataset size between these sets and HapMap SNPs. Click here for file
