# Supplementary material for: Genomic features defining exonic variants that modulate splicing
Source: Genome Biol. 2010 Feb 16;11(2):R20. doi: 10.1186/gb-2010-11-2-r20 (PMC2872880; doi:10.1186/gb-2010-11-2-r20)
Supplement: Additional file 1 — List of 87 synonymous and missense splice-affecting genome variants (SAVs) that cause exon skipping used for analysis in this study. The variants are derived from [12,13,37,41,44,47,48,54-56,65-103]. [file gb-2010-11-2-r20-S1.pdf]

Table S1 – List of 87 Synonymous and Missense variants that cause exon skipping used for analysis in this study. The variants are located in 40 genes and 47 individual exons. Variant locations are from human genome assembly hg18. References can be found in the main text of the paper.

SNP No.	Chr	Position	Gene	Potential Coding effect	Variant (Protein)	Variant (DNA)	Reference
1	1	196932540	**PTPRC**	Synonymous	P->P	C->G	[55]
2	2	211181402	**CPS1**	Synonymous	S->S	C->A	[64]
3	3	37017458	**MLH1**	Synonymous	D->D	T->C	[65]
4	3	143757998	**ATR**	Synonymous	E->E	A->G	[37]
5	4	185806724	**CASP3**	Synonymous (5′ UTR)	-	G->A	[53]
6	5	69408148	**SMN1**	Synonymous	L->L	A->G	[43]
7	5	70283529	**SMN2**	Synonymous	F->F	C->T	[66]
8	5	112198672	**APC**	Synonymous	R->R	G->T	[67]
9	7	117017661	**CFTR**	Synonymous	A->A	T->A	[46]
10	7	117017667	**CFTR**	Synonymous	L->L	G->A	[46]
11	7	117017682	**CFTR**	Synonymous	S->S	T->C	[46]
12	7	117017691	**CFTR**	Synonymous	G->G	A->G	[46]
13	7	117017691	**CFTR**	Synonymous	G->G	A->T	[46]
14	7	117017694	**CFTR**	Synonymous	Y->Y	C->T	[46]
15	10	42929995	**RET**	Synonymous	I->I	C->T	[68]
16	10	114190345	**ZDHHC6**	Synonymous	T->T	C->T	[53]
17	11	118464207	**HMBS**	Synonymous	R->R	C->G	[69]
18	14	22444702	**RBM23**	Synonymous	R->R	G->A	[53]
19	15	46516836	**FBN1**	Synonymous	I->I	C->T	[70]
20	15	78251635	**FAH**	Synonymous	N->N	C->T	[71]
21	17	26551622	**NF1**	Synonymous	Q->Q	G->A	[72]
22	17	26689883	**NF1**	Synonymous	Y->Y	C->T	[73]
23	17	59349223	**GH1**	Synonymous	E->E	A->G	[13]
24	19	11088602	**LDLR**	Synonymous	N->N	C->T	[54]
25	20	44185176	**CD40**	Synonymous	T->T	A->T	[74]
26	X	19281185	**PDHA**	Synonymous	Y->Y	C->T	[12]
27	X	19281200	**PDHA**	Synonymous	I->I	C->T	[12]
28	X	19282574	**PDHA**	Synonymous	G->G	A->G	[75]
29	X	32276456	**DMD**	Synonymous	F->F	C->T	[76]
30	X	40341465	**XMRE**	Synonymous	D->D	C->T	[77]
31	X	53475492	**HADH2**	Synonymous	R->R	C->A	[78]
32	X	133460368	**HPRT1**	Synonymous	F->F	C->T	[79]
33	1	75971876	**ACADM**	Missense	T->I	C->T	[80]
34	2	48774922	**LHCGR**	Missense	E->K	G->A	[81]
35	3	37017456	**MLH1**	Missense	N->H	G->C	[65]
36	3	37017456	**MLH1**	Missense	N->Y	G->T	[65]
37	3	37065091	**MLH1**	Missense	R->P	G->C	[82]

38	3	37065091	**MLH1**	Missense	R->L	G->T	[82]
39	4	104025813	**CISD2**	Missense	E->G	G->C	[83]
40	5	69408148	**SMN1**	Missense	L->F	A->C	[43]
41	5	69408149	**SMN1**	Missense	N->H	A->C	[43]
42	5	69408152	**SMN1**	Missense	X->E	T->G	[43]
43	5	69408154	**SMN1**	Missense	X->Y	A->T	[43]
44	5	69408154	**SMN1**	Missense	X->Y	A->C	[43]
45	5	74052072	**HEXB**	Missense	D->G	A->G	[40]
46	7	117017657	**CFTR**	Missense	D->G	A->G	[47]
47	10	124802603	**ACADSB**	Missense	M->V	A->G	[84]
48	11	107692006	**ATM**	Missense	E->K	G->A	[85]
49	13	31835504	**BRCA2**	Missense	T->R	C->G	[86]
50	13	47851743	**RB1**	Missense	G->R	G->A	[87]
51	15	38487132	**IVD**	Missense	R->C	C->T	[88]
52	15	38487133	**IVD**	Missense	R->P	G->T	[88]
53	15	38487189	**IVD**	Missense	D->N	G->A	[88]
54	16	8812504	**PMM2**	Missense	E->K	G->A	[89]
55	17	4745668	**CHRNE**	Missense	F->V	T->G	[90]
56	17	26521112	**NF1**	Missense	D->V	A->T	[91]
57	17	26551623	**NF1**	Missense	L->M	C->A	[72]
58	17	38469489	**BRCA1**	Missense	E->K	G->A	[92]
59	17	59349200	**GH1**	Missense	K->R	A->G	[93]
60	17	59349202	**GH1**	Missense	Q->H	G->T	[93]
61	17	59349205	**GH1**	Missense	E->D	A->T	[93]
62	17	59349206	**GH1**	Missense	E->V	A->T	[93]
63	17	59349223	**GH1**	Missense	E->D	A->C	[13]
64	17	59349224	**GH1**	Missense	E->G	A->G	[13]
65	17	59349225	**GH1**	Missense	E->Q	G->C	[13]
66	20	42685097	**ADA**	Missense	A->T	G->A	[94]
67	X	19282542	**PDHA1**	Missense	A->T	G->A	[95]
68	X	19282611	**PDHA1**	Missense	A->T	G->A	[96]
69	X	32276455	**DMD**	Missense	N->Y	A->T	[76]
70	X	32276456	**DMD**	Missense	F->L	C->G	[76]
71	X	32276456	**DMD**	Missense	F->L	C->A	[76]
72	X	32276457	**DMD**	Missense	F->Y	T->A	[76]
73	X	32276458	**DMD**	Missense	F->I	T->A	[76]
74	X	77184841	**ATP7A**	Missense	G->R	G->A	[97]
75	X	102928267	**PLP1**	Missense	R->W	C->T	[98]
76	X	102928267	**PLP1**	Missense	R->G	C->G	[98]
77	X	133435146	**HPRT1**	Missense	G->V	G->T	[99]
78	X	133436885	**HPRT1**	Missense	R->H	G->A	[99]
79	X	133455283	**HPRT1**	Missense	A->E	C->A	[99]
80	X	133460309	**HPRT1**	Missense	G->R	G->A	[100]
81	X	133460310	**HPRT1**	Missense	G->V	G->T	[100]
82	X	133460315	**HPRT1**	Missense	E->K	G->A	[99]

83	X	133460322	**HPRT1**	Missense	P->L	C->T	[79]
84	X	133460351	**HPRT1**	Missense	D->Y	G->T	[99]
85	X	133460361	**HPRT1**	Missense	E->V	A->T	[99]
86	X	133460373	**HPRT1**	Missense	D->V	A->T	[101]
87	X	153783589	**F8**	Missense	R->W	C->T	[102]
