# Supplementary material for: Genomic features defining exonic variants that modulate splicing
Source: Genome Biol. 2010 Feb 16;11(2):R20. doi: 10.1186/gb-2010-11-2-r20 (PMC2872880; doi:10.1186/gb-2010-11-2-r20)
Supplement: Additional file 2 — List of 80 synonymous and missense variants that have been experimentally tested in mini-gene constructs and do not cause changes in splicing. The variants are derived from [74,104-106]. [file gb-2010-11-2-r20-S2.pdf]

Table S4 – List of 80 Synonymous and Missense variants that have been experimentally tested in mini-gene constructs and do not cause changes in splicing. This group consists of variants that occur both naturally (i.e. SNPs) [152] or have been created artificially by mutagenesis [73,103,153]. References are in the main text of the paper.

SNP No.	Chr	Position	Gene	Potential Coding Effect	Mutation (Protein)	Mutation (DNA)	Reference
1	21	21668058	**NCAM2**	Missense	L->P	T->C	[152]
2	5	13882799	**DNAH5**	Synonymous	A->A	C->T	[152]
3	12	103233689	**TXNRD1**	Synonymous	L->L	C->T	[152]
4	1	181451239	**LAMC2**	Synonymous	S->S	C->T	[152]
5	1	27552384	**SYTL1**	Missense	Q->R	A->G	[152]
6	2	98516378	**INPP4A**	Synonymous	T->T	G->A	[152]
7	2	24378462	**ITSN2**	Missense	V->I	G->A	[152]
8	12	27758994	**MRPS35**	Missense	G->R	G->A	[152]
9	4	9531265	**SLC2A9**	Missense	V->I	G->A	[152]
10	4	96325345	**UNC5C**	Missense	M->T	T->C	[152]
11	15	84607033	**AGBL1**	Synonymous	D->D	C->T	[152]
12	1	17174367	**MFAP2**	Synonymous	H->H	T->C	[152]
13	2	218976025	**CTDSP1**	Synonymous	H->H	C->T	[152]
14	16	83480360	**CRISPLD2**	Synonymous	A->A	C->T	[152]
15	18	75574115	**CTDP1**	Missense	T->M	C->T	[152]
16	5	38406294	**EGFLAM**	Missense	W->R	T->C	[152]
17	12	108102248	**ACACB**	Synonymous	D->D	T->C	[152]
18	22	29187448	**SEC14L3**	Synonymous	D->D	T->C	[152]
19	12	116154297	**NOS1**	Synonymous	D->D	C->T	[152]
20	11	68462250	**IGHMBP2**	Missense	T->K	C->A	[152]
21	2	160516321	**PLA2R1**	Missense	G->S	G->A	[152]
22	7	158520087	**VIPR2**	Synonymous	N->N	C->T	[152]
23	7	21565758	**DNAH11**	Synonymous	N->N	C->T	[152]
24	17	68922486	**SDK2**	Synonymous	T->T	G->A	[152]
25	10	102255805	**SEC31B**	Synonymous	H->H	T->C	[152]
26	17	70121684	**CD300E**	Missense	G->R	G->A	[152]
27	15	39953792	**SPTBN5**	Missense	R->H	G->A	[152]
28	4	154733172	**KIAA0922**	Synonymous	H->H	C->T	[152]
29	3	133701313	**DNAJC13**	Missense	A->S	G->T	[152]
30	2	200962201	**LOC26010**	Synonymous (5' UTR)	NA	A->G	[152]
31	5	41036100	**AC114967.2**	Synonymous	Y->Y	C->T	[152]
32	12	47351994	**C12orf41**	Synonymous	T->T	A->G	[152]
33	10	113925369	**GPAM**	Missense	E->G	A->G	[152]
34	3	113403806	**SLC9A10**	Missense	S->I	G->T	[152]
35	3	155501581	**DHX36**	Synonymous	S->S	G->C	[152]
36	4	87925530	**PTPN13**	Missense	Y->D	T->G	[152]
37	15	38443137	**DISP2**	Missense	P->A	C->G	[152]
38	9	83456967	**TLE1**	Synonymous	E->E	A->G	[152]
39	X	14797068	**FANCB**	Synonymous (5' UTR)	NA	C->G	[152]
40	6	41106119	**UNC5CL**	Synonymous	C->C	C->T	[152]
41	11	60422917	**PRPF19**	Synonymous	S->S	C->T	[152]
42	11	85646271	**EED**	Synonymous	L->L	C->T	[152]
43	3	37042476	**MLH1**	Missense	K->N	G->T	[153]
44	3	37042191	**MLH1**	Synonymous	L->L	G->T	[153]
45	3	37042232	**MLH1**	Synonymous	A->A	C->T	[153]
46	3	37042239	**MLH1**	Missense	Q->H	G->C	[153]
47	3	37042240	**MLH1**	Missense	M->L	A->T	[153]
48	3	37042297	**MLH1**	Missense	K->Q	A->C	[153]
49	3	37042335	**MLH1**	Missense	E->D	G->C	[153]
50	3	37042363	**MLH1**	Missense	A->S	G->C	[153]
51	3	37042376	**MLH1**	Missense	D->V	A->T	[153]

52	3	37042406	**MLH1**	Missense	A->V	C->T	[153]
53	3	37042432	**MLH1**	Synonymous	L->L	T->C	[153]
54	3	37042454	**MLH1**	Missense	G->G	G->C	[153]
55	7	116975945	**CFTR**	Missense	L->F	A->C	[103]
56	7	116975973	**CFTR**	Missense	N->D	A->G	[103]
57	7	116976002	**CFTR**	Missense	S->R	C->A	[103]
58	7	116976048	**CFTR**	Missense	D->N	G->A	[103]
59	7	116976081	**CFTR**	Missense	L->M	T->A	[103]
60	7	116976081	**CFTR**	Synonymous	L->L	T->C	[103]
61	7	116976083	**CFTR**	Missense	L->F	G->C	[103]
62	7	116976087	**CFTR**	Missense	V->F	G->T	[103]
63	17	26689881	**NF1**	Missense	Y->N	T->A	[73]
64	17	26689881	**NF1**	Missense	Y->H	T->C	[73]
65	17	26689881	**NF1**	Missense	Y->D	T->G	[73]
66	17	26689882	**NF1**	Missense	Y->S	A->C	[73]
67	17	26689882	**NF1**	Missense	Y->C	A->G	[73]
68	17	26689882	**NF1**	Missense	Y->F	A->T	[73]
69	17	26689884	**NF1**	Missense	N->H	A->C	[73]
70	17	26689884	**NF1**	Missense	N->D	A->G	[73]
71	17	26689884	**NF1**	Missense	N->Y	A->T	[73]
72	17	26689885	**NF1**	Missense	N->T	A->C	[73]
73	17	26689885	**NF1**	Missense	N->S	A->G	[73]
74	17	26689885	**NF1**	Missense	N->I	A->T	[73]
75	17	26689887	**NF1**	Missense	S->R	A->C	[73]
76	17	26689887	**NF1**	Missense	S->G	A->G	[73]
77	17	26689887	**NF1**	Missense	S->C	A->T	[73]
78	17	26689888	**NF1**	Missense	S->N	G->A	[73]
79	17	26689888	**NF1**	Missense	S->I	G->T	[73]
80	17	26689888	**NF1**	Missense	S->T	G->C	[73]
