# Supplementary material for: Genomic features defining exonic variants that modulate splicing
Source: Genome Biol. 2010 Feb 16;11(2):R20. doi: 10.1186/gb-2010-11-2-r20 (PMC2872880; doi:10.1186/gb-2010-11-2-r20)
Supplement: Additional file 3 — (a) Full results of ESR changes and bootstrap analysis of exon skipping SAVs vs. hSNPs, (b) splice-neutral variants vs. hSNPs, (c) SAVs that cause exon inclusion vs. hSNPs, (d) ectopic SAVs vs. hSNPs and (e) ectopic-like hSNPs vs. hSNPs with a ΔSS of 0. [file gb-2010-11-2-r20-S3.pdf]

Table S5a. Results of bootstrap analysis of exon skipping SAVs vs. HapMap SNPs for changes in seven ESR prediction sets.

ESR change type	Total number of SAVs with changes[1]			Total number of ESR changes from SAVs[2]		
	No. SAVs with change	Z-score*	p-value**	Total changes	Z-score*	p-value**
ESE-finder lost	39	-0.256	0.797	54	-0.162	0.871
ESE-finder gained	31	-1.375	0.168	39	-1.619	0.105
NI-ESE lost	55	3.901	9.56×10^{-5}	138	6.316	2.68×10^{-10}
NI-ESE altered	35	-2.603	0.0092	83	-1.534	0.124
NI-ESE gained	20	-3.611	3.0×10^{-4}	32	-3.346	8.1×10^{-4}
NI-ESS lost	7	-1.951	0.050	17	-0.824	0.409
NI-ESS altered	15	1.667	0.095	22	0.997	0.318
NI-ESS gained	43	6.232	4.59×10^{-10}	98	8.498	1.92×10^{-17}
RESCUE-ESE lost	36	4.847	1.25×10^{-6}	70	5.963	2.46×10^{-9}
RESCUE-ESE altered	14	1.121	0.262	23	1.242	0.213
RESCUE-ESE gained	14	-1.619	0.105	21	-1.490	0.136
Fas-Hex2 ESS lost	3	-1.811	0.070	5	-1.338	0.180
Fas-Hex2 ESS altered	5	1.759	0.078	7	1.899	0.057
Fas-Hex2 ESS gained	26	5.608	2.04×10^{-8}	47	7.334	2.23×10^{-13}
Ast ESRs lost	37	1.962	0.049	50	2.803	0.00505
Ast ESRs altered	13	-0.213	0.831	15	0.073	0.941
Ast ESRs gained	20	-2.521	0.011	23	-2.416	0.0156
PESEs lost	29	2.165	0.030	53	3.032	0.00242
PESEs altered	7	0.005	0.995	10	0.038	0.9691
PESE gained	9	-2.795	0.0051	14	-2.280	0.0226
PESS lost	6	0.774	0.438	6	-0.002	0.998
PESS altered	4	2.823	0.00475	6	3.055	0.00224
PESS gained	13	3.154	0.0016	20	3.893	9.89×10^{-5}
Comp-ESE lost	41	2.841	0.0044	94	5.230	1.69×10^{-7}
Comp-ESE altered	19	-2.721	0.0065	31	-2.307	0.021
Comp-ESE gained	19	0.170	0.864	34	0.630	0.528
Comp-ESS lost	2	-2.463	0.013	4	-1.908	0.0563
Comp-ESS altered	2	-0.687	0.491	5	0.370	0.711
Comp-ESS gained	30	5.773	7.77×10^{-9}	61	8.362	3.84×10^{-16}

*The Z score represents the tail of the comparison (i.e. negative Z scores indicate SAVs have a smaller values than expected and positive Z score indicate SAVs have a larger value than expected

**P-values associated with the Z-scores are shown. All p-values considered significant (≤ 0.05) are highlighted in yellow.
[1] Total number of SAVs that have a change of this type out of a total of 87 SAVs
[2] Total number of changes of that type from the entire set of 87 SAVs.

Table S5b. Results of bootstrap analysis of experimentally verified splice-neutral variants (from Table S4) vs. hSNPs for ESR changes using the Neighborhood Inference set of ESRs.

ESR change type	Total number of SAVs with changes[1]			Total number of ESR changes from SAVs[2]		
	No. SAVs with change	Z-score*	p-value**	Total changes	Z-score*	p-value**
**NI-ESE lost**	37	0.738	0.460	79	1.547	0.121
**NI-ESE altered**	55	1.094	0.27	99	1.600	0.110
**NI-ESE gained**	31	-0.483	0.628	63	0.111	0.911
**NI-ESS lost**	10	-0.745	0.455	18	-0.378	0.704
**NI-ESS altered**	15	-1.450	0.146	30	-1.429	0.152
**NI-ESS gained**	5	-0.634	0.5255	7	0.090	0.928

[1] Total number of SAVs that have a change of this type out of a total of 80 splice-neutral variants
[2] Total number of changes of that type from the entire set of 80 splice-neutral variants.

Table S5c. Results of bootstrap analysis of SAVs causing increased exon *inclusion* vs. hSNPs for ESR changes using the Neighborhood Inference (NI) set of ESRs.

ESR change type	Total number of SAVs with changes[1]			Total number of ESR changes from SAVs[2]		
	No. SAVs with change	Z-score*	p-value**	Total changes	Z-score*	p-value**
**NI-ESE lost**	3	-2.488	0.0128	5	-2.063	0.039
**NI-ESE altered**	13	2.064	0.0389	25	1.753	0.0794
**NI-ESE gained**	11	0.0631	0.949	18	-0.917	0.358
**NI-ESS lost**	12	4.986	6.15×10^{-7}	21	5.427	5.71×10^{-8}
**NI-ESS altered**	6	2.562	0.0103	7	1.158	0.246
**NI-ESS gained**	2	-1.279	0.2	3	-1.180	0.237

[1] Total number of SAVs that have a change of this type out of a total of 20 inclusion SAVs.
[2] Total number of changes of that type from the entire set of 20 inclusion SAVs.

Table S5d. Results of bootstrap analysis of 54 ectopic SAVs vs. ~14,500 hSNPs (with ΔSS scores of 0) for ESR changes using the Neighborhood Inference set of ESRs.

ESR change type	Total number of SAVs with changes[1]			Total number of ESR changes from SAVs[2]		
	No. SAV with change	Z-score*	p-value**	Total changes	Z-score*	p-value**
NI-ESE lost	22	-0.195	0.845	43	0.067	0.945
NI-ESE altered	17	-3.170	0.00152	31	-2.991	0.00277
NI-ESE gained	22	0	0.999	42	0.121	0.903
NI-ESS lost	9	0.320	0.748	13	-0.107	0.914
NI-ESS altered	12	2.653	0.00796	23	3.096	0.00195
NI-ESS gained	30	6.045	$1.49x10^{-9}$	68	7.897	$2.85x10^{-15}$

[1] Total number of SAVs that have a change of this type out of a total of 54 ectopic SAVs.
[2] Total number of changes of that type from the entire set of 54 ectopic SAVs.

Table S5e. Results of bootstrap analysis of the top 54 hSNPs with greatest *ΔSS* score *(ΔSS* > 7.5) that create a de novo ectopic splice site with a greater score than the natural site vs. ~14,500 hSNPs (with ΔSS scores of 0) for ESR changes using the Neighborhood Inference set of ESRs.

ESR change type	Total number of SAVs with changes[1]			Total number of ESR changes from SAVs[2]		
	No. SAV with change	Z-score*	p-value**	Total changes	Z-score*	p-value**
NI-ESE lost	26	0.714	0.474	58	1.654	0.0980
NI-ESE altered	27	-0.805	0.420	44	-2.047	0.0406
NI-ESE gained	11	-3.333	0.000856	19	-2.811	0.00493
NI-ESS lost	6	-0.987	0.323	9	-1.017	0.309
NI-ESS altered	8	0.857	0.390	11	0.312	0.754
NI-ESS gained	15	0.982	0.325	32	1.885	0.0594

[1] Total number of SAVs that have a change of this type out of a total of 54 ectopic-like hSNPs.
[2] Total number of changes of that type from the entire SAV set.
