# Supplementary material for: Genomic features defining exonic variants that modulate splicing
Source: Genome Biol. 2010 Feb 16;11(2):R20. doi: 10.1186/gb-2010-11-2-r20 (PMC2872880; doi:10.1186/gb-2010-11-2-r20)
Supplement: Additional file 4 — Set of graphs illustrating that exon-skipping SAVs are significantly overrepresented within certain positions across the four ESEfinder matrices. [file gb-2010-11-2-r20-S4.pdf]

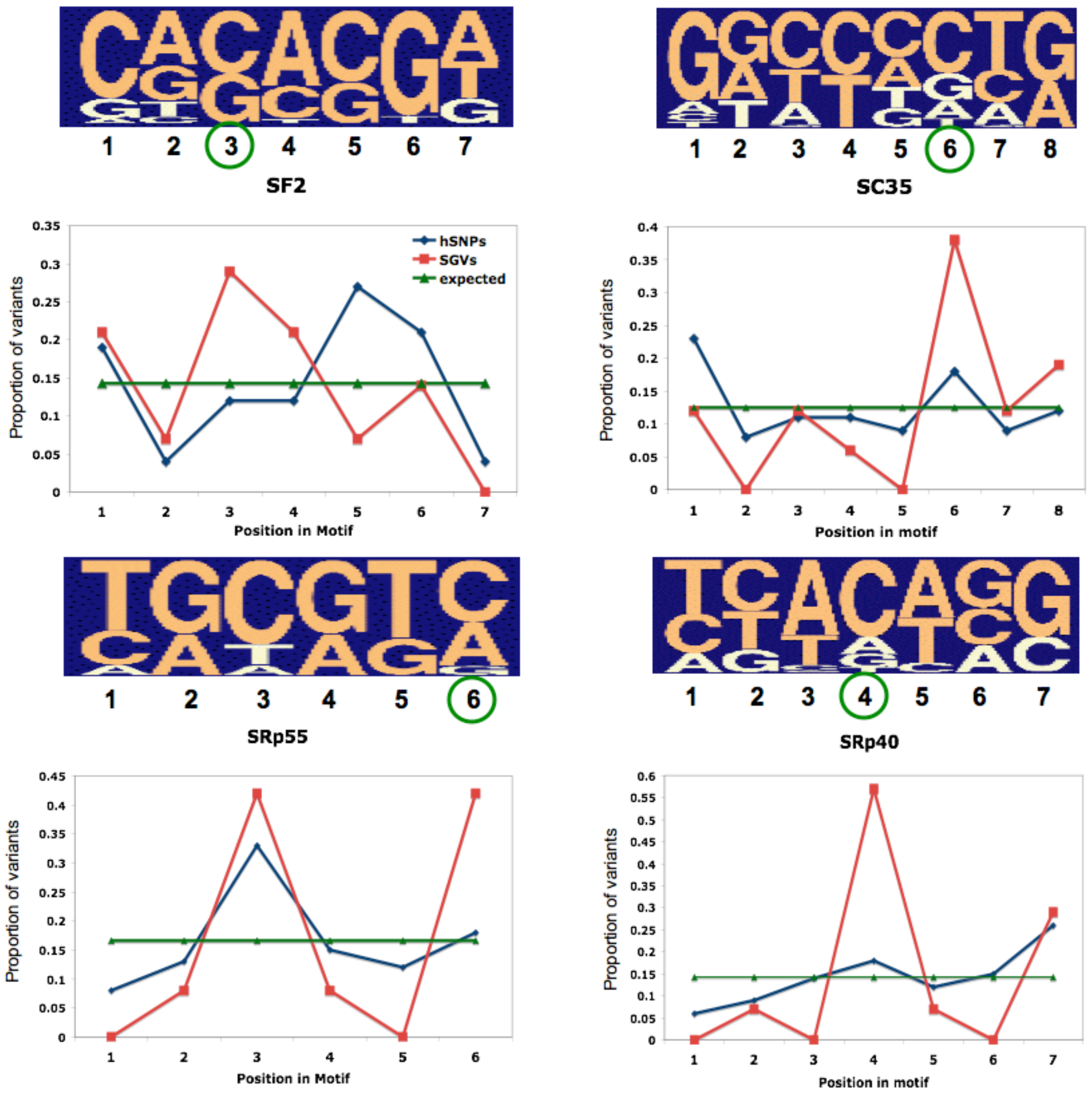

Figure S2. Proportion of SAVs and hSNPs that destroy an ESEfinder motif and the position in which they occur across four binding sites. Positions that were significantly overrepresented in SAVs compared to hSNPs by χ^2 test are circled in green (SF2-3, P=0.0322; SC35-6, P=0.0371; SRp55-6, P=0.0396; SRp40-4, P=1x10^{-3}). A uniform 'expected' distribution of SNPs across all positions is displayed for comparison as a green line. Pictograms representing the consensus nucleotide at each position of the binding site were adapted from Cartegni et al. [58].
