# Supplementary material for: Genomic features defining exonic variants that modulate splicing
Source: Genome Biol. 2010 Feb 16;11(2):R20. doi: 10.1186/gb-2010-11-2-r20 (PMC2872880; doi:10.1186/gb-2010-11-2-r20)
Supplement: Additional file 5 — Methods and results for an analysis on whether using local RNA secondary structure as a filter improves our ability to distinguish exon skipping SAVs from hSNPs. Our results suggest that using this filter does not improve our ability to predict SAVs although a small number of SAVs may arise from the indirect uncovering of ESS motifs by changes in local RNA secondary structure. [file gb-2010-11-2-r20-S5.pdf]

Methods and Results S1.

Local RNA secondary structure analysis

Using the approach set out in [22] we investigated whether modulation of local RNA secondary structure and its effect on the activity of ESEs and ESS was an important feature in SAVs and could further differentiate them from hSNPs. In this approach, there are two ways in which a genomic variant may affect splicing regulatory elements via local RNA secondary structure. The first is by destroying ESEs or creating ESSs located in 'exposed' single-stranded regions (which we will refer to as the 'direct' mechanism), and the second is by modulating the local RNA structure in a way that moves active ESE motifs that were single stranded into hidden stem structures and/or moving hidden ESSs into active loop structures (referred to as the 'indirect' mechanism) (see Methods and Diagram below).

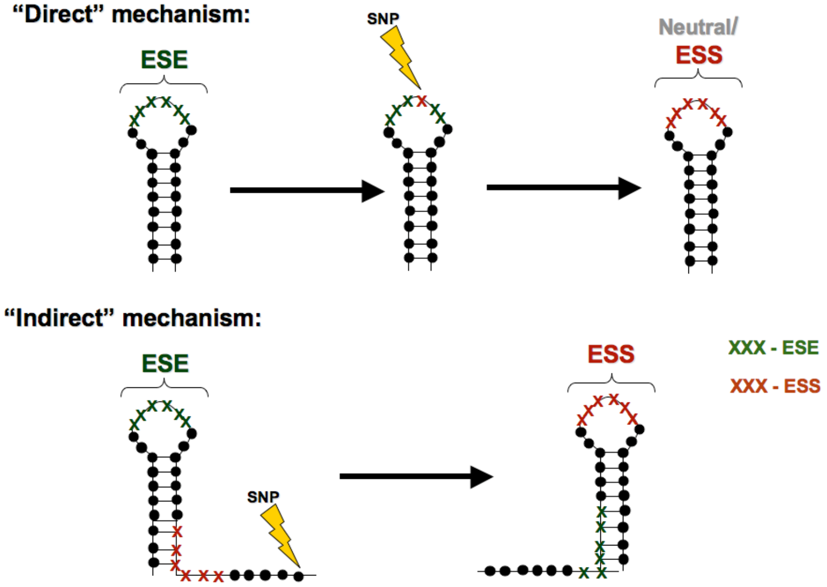

Summing the changes from both RNA-structure based changes for each variant, we compared ESE/ESS loss and gain for the SAV and hSNP sets using NI-ESRs. We found that ESE losses via changes in RNA secondary structure were very similar between SAVs and hSNPs (P=0.309). Similarly, the extent of ESE losses (i.e. predominance of 2 or more changes) was also comparable between SAVs and hSNPs (P=0.171). In contrast, the difference in the proportion of

SAVs with ESS gains compared to hSNPs remained significant (P=0.0015) despite being found in less than a quarter of SAVs, as well as the extent of ESS gains ($P=1.13x10^{-5}$). This is a substantial reduction in the differential from just using the primary sequence ($P=9.6x10^{-5}$ and $P=1.92x10^{-17}$ respectively). Interestingly, when we looked at the significance of ESS gains from each mechanism in isolation, those from the 'indirect' mechanism are the major contributor to this significance ($P=8.8x10^{-5}$) rather than the 'direct' mechanism (P=0.011). It is therefore possible that a small subset of SAVs do indeed function via changed structural accessibility of splicing silencer elements. Alternatively, as the 'indirect' mechanism is largely dependant on the presence of preexisting ESSs in the exon, the signal we see may just be an artifact of the greater prevalence of ESSs in SAV exons. Further evidence is therefore needed to confirm whether RNA secondary structure is indeed a relevant mechanism in these cases. Nevertheless, local RNA secondary structure (as implemented by this method) does not go very far in explaining the ~30% (26/87) of SAVs that exhibited no change in ESEs or ESSs in the original analysis, as only three (3.5%) of these have any RNA-secondary structure based changes in ESEs/ESSs (two in CFTR and one in SMN1). In summary, while local RNA secondary structure may play important roles in certain cases, it does not appear to play a predominant role in our set of SAVs and that this computational implementation of structure prediction is unable to strongly differentiate splice-affecting variants from splicing-neutral polymorphisms.

Methods for local RNA secondary structure analysis

Two types of changes relating to RNA secondary structure using the NI set of ESRs were measured: 1.) whether a SNP caused an ESE loss or ESS gain in a hexamer that was considered 'single-stranded' or in an open conformation and referred to as the 'direct' mechanism and 2.) whether a SNP alters the RNA secondary structure across the exon in a way that changes open ESEs to a more hidden conformation and/or changes a closed ESS to a more 'exposed' conformation and referred to as the 'indirect' mechanism. Both approaches were carried out using

methods previously described in [22]. Briefly, for the 'direct' mechanism, a measurement of single-strandedness, the mean PU (probability of unpaired) value, was calculated for each overlapping wild type or ancestral hexamer in which a variant plays a role. This value is defined as

$$MeanPU = \frac{\sum_{i=11}^{30} e^{\frac{Eall^i - Eunpaired^i}{RT}}}{20}$$

where i is the length of flanking sequence on either side of the hexamer which ranges from 11-30bp, $Eall^i$ is the free energy of the ensemble of all structures with flanking sequence of length i, $Eunpaired^i$ is the free energy of the ensemble of all structures that have the complete hexamer unpaired with flank of length i, R is the universal gas constant and T is the temperature in Kelvin. $Eall^i$ and $Eunpaired^i$ are calculated using the partition function version of the program RNAfold with unpaired constraints introduced using parameter –C. As PU values are influenced by GC content and the size of the motif under study [22], an appropriate threshold PU value (for a hexamer to be considered single-stranded) was calculated by creating a distribution of 120 meanPU values from randomly shuffled sequences with the same dinucleotide content as the sequence used to calculate meanPU using the program *dishuffle*. We used the mean of this distribution as the threshold value.

For the 'indirect' mechanism, for each SAV/HapMap exon, mean PU values were calculated for all overlapping hexamers across the exon for wild type and variant sequences of the exon using the method described above. A value, ΔPU (change in single strandedness), was calculated by subtracting the PU value for a hexamer from the variant exon from the PU value for the same hexamer in the wild-type exon. Any hexamer with a negative $\Delta PU \leq -0.25$ (the hexamer has become more single stranded) and is designated as an ESE/ESS in the variant sequence is counted as an ESE/ESS gain. A hexamer with a positive $\Delta PU \geq 0.25$ (the hexamer has become

less single stranded) is counted as an ESE/ESS loss. Different thresholds varying from 0.25-0.4 in 0.05 increments were also used, with a concomitant decrease in discrimination of SAVs from hSNPs as the threshold was increased.
