# Supplementary material for: Genomic features defining exonic variants that modulate splicing
Source: Genome Biol. 2010 Feb 16;11(2):R20. doi: 10.1186/gb-2010-11-2-r20 (PMC2872880; doi:10.1186/gb-2010-11-2-r20)
Supplement: Additional file 6 — List of 20 variants that cause increased exon inclusion. The variants are derived from [44,47,104,107,108]. [file gb-2010-11-2-r20-S6.pdf]

Table S2 – List of 20 variants that cause increased exon inclusion

All variants were taken from the Alternative Splicing Mutation Database [26] as variants that have an SE value > 0. References can be found in the main text of the paper.

SNP	Chr	Position	Gene	Coding effect	Variant (Protein)	Variant (DNA)	Reference
1	5	70283543	**SMN1**	Missense	K->I	A->T	[43]
2	5	70283560	**SMN1**	Missense	H->T	C->T	[43]
3	5	70283570	**SMN1**	Missense	N->T	A->C	[43]
4	7	116975948	**CFTR**	Missense	F->L	T->G	[103]
5	7	116976075	**CFTR**	Missense	Q->G	C->G	[103]
6	7	116976075	**CFTR**	Missense	Q->L	C->A	[103]
7	7	116976076	**CFTR**	Missense	Q->R	A->G	[103]
8	7	116976076	**CFTR**	Missense	Q->L	A->T	[103]
9	7	116976076	**CFTR**	Missense	Q->P	A->C	[103]
10	7	116976077	**CFTR**	Missense	Q->H	G->T	[103]
11	7	116976077	**CFTR**	Missense	Q->H	G->C	[103]
12	7	116976078	**CFTR**	Synonymous	L->L	T->C	[103]
13	7	116976078	**CFTR**	Missense	L->M	T->A	[103]
14	7	117017676	**CFTR**	Synonymous	L->L	A->G	[46]
15	7	117017679	**CFTR**	Synonymous	N->N	C->T	[46]
16	7	117017682	**CFTR**	Synonymous	S->S	T->G	[46]
17	7	117017685	**CFTR**	Synonymous	P->P	T->A	[46]
18	17	41443542	**MAPT**	Synonymous	L->L	T->C	[104]
19	17	41443527	**MAPT**	Missense	N->K	T->G	[104]
20	17	41443578	**MAPT**	Synonymous	N->N	T->C	[105]
