# Supplementary material for: Genomic features defining exonic variants that modulate splicing
Source: Genome Biol. 2010 Feb 16;11(2):R20. doi: 10.1186/gb-2010-11-2-r20 (PMC2872880; doi:10.1186/gb-2010-11-2-r20)
Supplement: Additional file 7 — Two plots that show that mean RC score is negatively correlated with minimum distance from a splice junction (top) but not correlated with exon length (bottom). [file gb-2010-11-2-r20-S7.pdf]

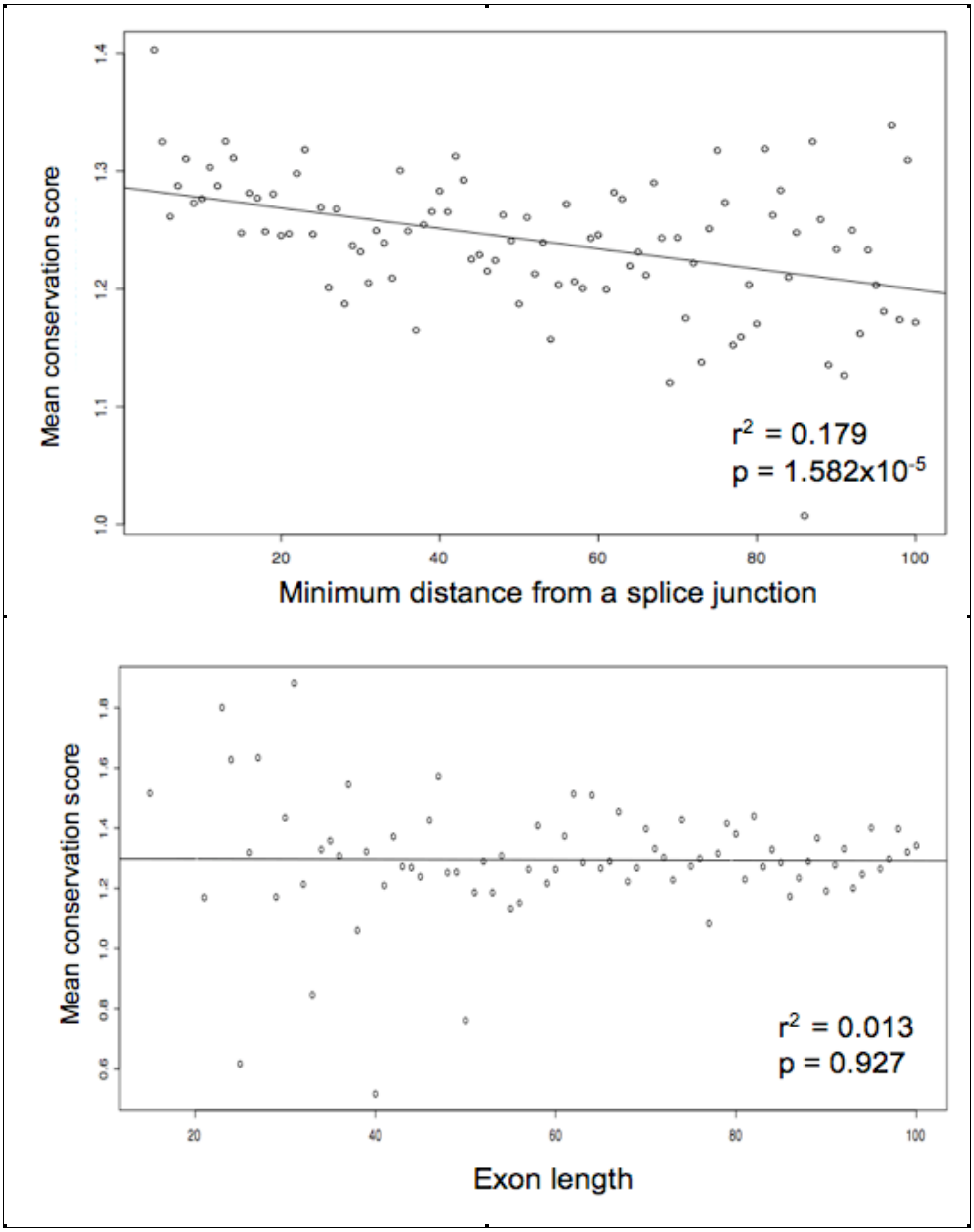

Figure S3. The regulatory constraint (RC) score is influenced by distance from the splice junction but not by exon length. Mean RC scores for hSNPs were plotted as a function of minimum distance from the splice junction (top) and exon length (bottom).
