# Supplementary material for: Genomic features defining exonic variants that modulate splicing
Source: Genome Biol. 2010 Feb 16;11(2):R20. doi: 10.1186/gb-2010-11-2-r20 (PMC2872880; doi:10.1186/gb-2010-11-2-r20)
Supplement: Additional file 8 — Only distributions of exon lengths up to 600 bp were plotted for clarity. Genome-wide exons were divided into constitutively spliced (CE) and alternatively spliced (AS) as defined by the Hollywood database [36]. A fifth, expected set of exons represents a set of exon lengths we would expect given the average distribution of hSNPs across the genome and fits the real distribution of HapMap exons closely. [file gb-2010-11-2-r20-S8.pdf]

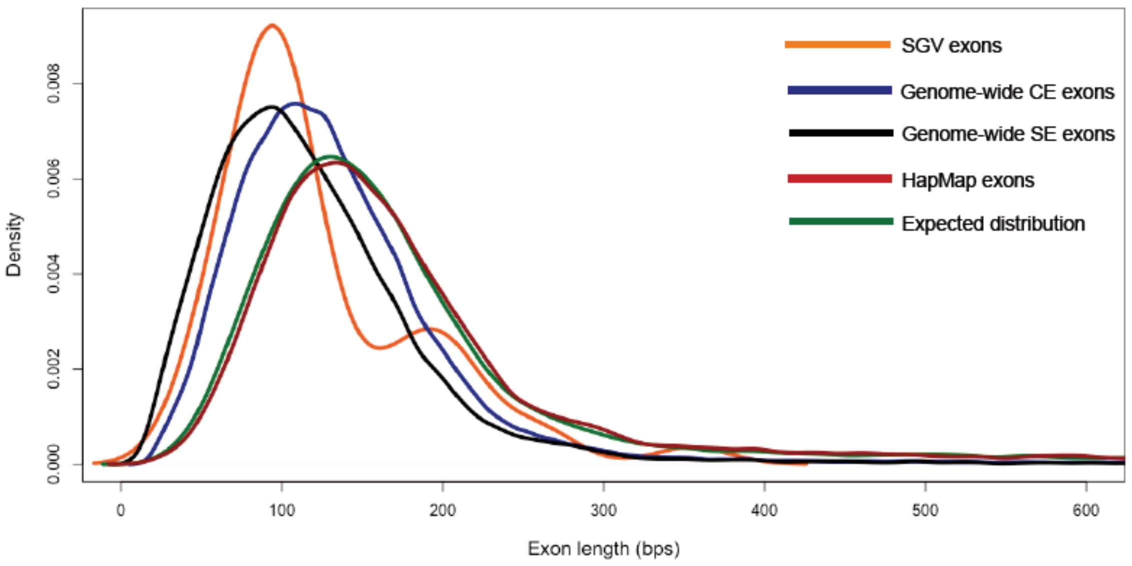


**Figure S4. Distribution of exon lengths for SAV exons versus HapMap and genome-wide exons.** Only distributions of exon lengths up to 600bp were plotted for clarity. Genome-wide exons were divided into constitutively spliced (CE) and alternatively spliced (AS) as defined by the Hollywood database. A fifth, expected set of exons represents a set of exon lengths we would expect given the average distribution of hSNPs across the genome and fits the real distribution of HapMap exons closely.
