# Supplementary material for: Genomic features defining exonic variants that modulate splicing
Source: Genome Biol. 2010 Feb 16;11(2):R20. doi: 10.1186/gb-2010-11-2-r20 (PMC2872880; doi:10.1186/gb-2010-11-2-r20)
Supplement: Additional file 9 — List of 54 variants that cause de novo 5' or 3' ectopic splice site activation. The variants are derived from [4,37,86,109-154]. [file gb-2010-11-2-r20-S9.pdf]

Table S3. List of 54 variants that cause de novo 5' or 3' ectopic splice site activation. Variants were obtained from DBASS5 and DBASS3. References can be found in the main text of the paper.

SNP No.	Chr	Position	Gene	Potential Coding effect	Splice site activation	Variant (DNA)	max(Δss)	Reference
1	3	15651990	**BTD**	Missense	3′	G->A	7.96	[106]
2	4	155708309	**FGB**	Missense	3′	T->A	8.37	[107]
3	6	31432182	**HLA-B**	Missense	3′	G->C	9.95	[108]
4	11	107511124	**ACAT1**	Missense	3′	C->T	2.11	[109]
5	15	41282727	**EPB42**	Missense	3′	G->T	13.31	[110]
6	19	17860206	**SLC5A5**	Missense	3′	C->G	8.06	[111]
7	X	32238252	**DMD**	Nonsense	3′	T->G	8.59	[112]
8	11	67567654	**TCIRG1**	Synonymous	3′	G->A	7.95	[113]
9	19	11084983	**LDLR**	Synonymous	3′	C->A	8.04	[114]
10	X	153838609	**F8**	Synonymous	3′	G->T	2.51	[115]
11	1	154375026	**LMNA**	Missense	5′	G->A	1.53	[4]
12	4	89215079	**PKD2**	Nonsense	5′	C->T	0	[116]
13	5	70980772	**MCCB**	Missense	5′	A->G	8.18	[117]
14	7	91702781	**KRIT1**	Missense	5′	C->G	1.81	[118]
15	7	91703738	**KRIT1**	Missense	5′	A->G	8.18	[118]
16	10	89682830	**PTEN**	Missense	5′	C->G	8.27	[119]
17	11	107708775	**ATM**	Missense	5′	C->T	7.75	[85]
18	16	67413594	**CDH1**	Missense	5′	C->T	7.75	[120]
19	17	26551666	**NF1**	Missense	5′	C->T	7.75	[121]
20	17	26580523	**NF1**	Missense	5′	G->A	3.81	[122]
21	17	26565668	**NF1**	Nonsense	5′	A->G	8.18	[122]
22	17	26583297	**NF1**	Missense	5′	T->A	5.33	[121]
23	17	38512021	**BRCA1**	Missense	5′	T->G	8.6	[123]
24	17	45619024	**COL1A1**	Missense	5′	A->G	8.18	[124]
25	X	100516810	**BTK**	Nonsense	5′	A->G	8.18	[125]
26	X	133436951	**HPRT1**	Missense	5′	G->T	7.65	[126]
27	X	135560190	**CD40L**	Nonsense	5′	G->T	4.23	[127]
28	X	148392770	**IDS**	Missense	5′	C->G	0	[128]
29	X	153235823	**FLNA**	Missense	5′	C->T	0	[129]
30	1	154375028	**LMNA**	Synonymous	5′	C->T	0.49	[130]
31	1	153474560	**GBA**	Synonymous	5′	A->G	8.18	[131]
32	1	156912581	**SPTA1**	Synonymous	5′	A->G	8.18	[132]
33	2	219382723	**CYP27A1**	Synonymous	5′	G->T	7.65	[133]
34	3	48593334	**COL7A1**	Synonymous	5′	C->T	7.75	[134]
35	3	143757971	**ATR**	Synonymous	5′	A->G	0	[37]
36	3	185449258	**ALG3**	Synonymous	5′	C->T	0.75	[135]
37	5	42747170	**GHR**	Synonymous	5′	C->T	7.76	[136]
38	5	42735837	**GHR**	Synonymous	5′	A->G	4.8	[137]
39	7	65070189	**GUSB**	Synonymous	5′	C->T	7.76	[138]

40	11	5204749	**HBB**	Synonymous	5′	G->A	2.24	[139]
41	11	5204753	**HBB**	Synonymous	5′	T->A	2.28	[140]
42	12	108497085	**MVK**	Synonymous	5′	C->T	7.75	[141]
43	12	46535733	**VDR**	Synonymous	5′	C->G	3.5	[142]
44	15	70428488	**HEXA**	Synonymous	5′	T->A	4.52	[143]
45	16	162980	**HBA2**	Synonymous	5′	C->T	7.76	[144]
46	16	66027759	**HSD11B2**	Synonymous	5′	C->G	2.51	[145]
47	17	42731797	**ITGB3**	Synonymous	5′	C->T	7.76	[146]
48	17	26580468	**NF1**	Synonymous	5′	G->A	3.51	[121]
49	17	26586877	**NF1**	Synonymous	5′	C->T	7.75	[121]
50	19	17808957	**JAK3**	Synonymous	5′	C->T	7.75	[147]
51	19	806755	**ELA2**	Synonymous	5′	C->A	7.03	[148]
52	X	100500293	**BTK**	Synonymous	5′	A->G	0	[149]
53	X	153301140	**TAZ**	Synonymous	5′	G->A	0	[150]
54	X	148376419	**IDS**	Synonymous	5′	C->T	7.75	[151]
