# Supplementary material for: Genomic features defining exonic variants that modulate splicing
Source: Genome Biol. 2010 Feb 16;11(2):R20. doi: 10.1186/gb-2010-11-2-r20 (PMC2872880; doi:10.1186/gb-2010-11-2-r20)
Supplement: Additional file 10 — Base substitution distributions in hSNPs were used a background nucleotide substitution rates in calculating an expected distribution of ESE/ESS changes of the NI-ESR hexamer set (Figure 3). Significant differences in distributions between skipping SAVs and hSNPs (such as that seen in A->T, T->C and G->T) and ectopic SAVs (A->T, A->C, T->A, C->G) while potentially of biological interest, should be treated with caution due to small number of skipping and ectopic SAVs and large discrepancy in dataset size between these sets and HapMap SNPs. [file gb-2010-11-2-r20-S10.pdf]

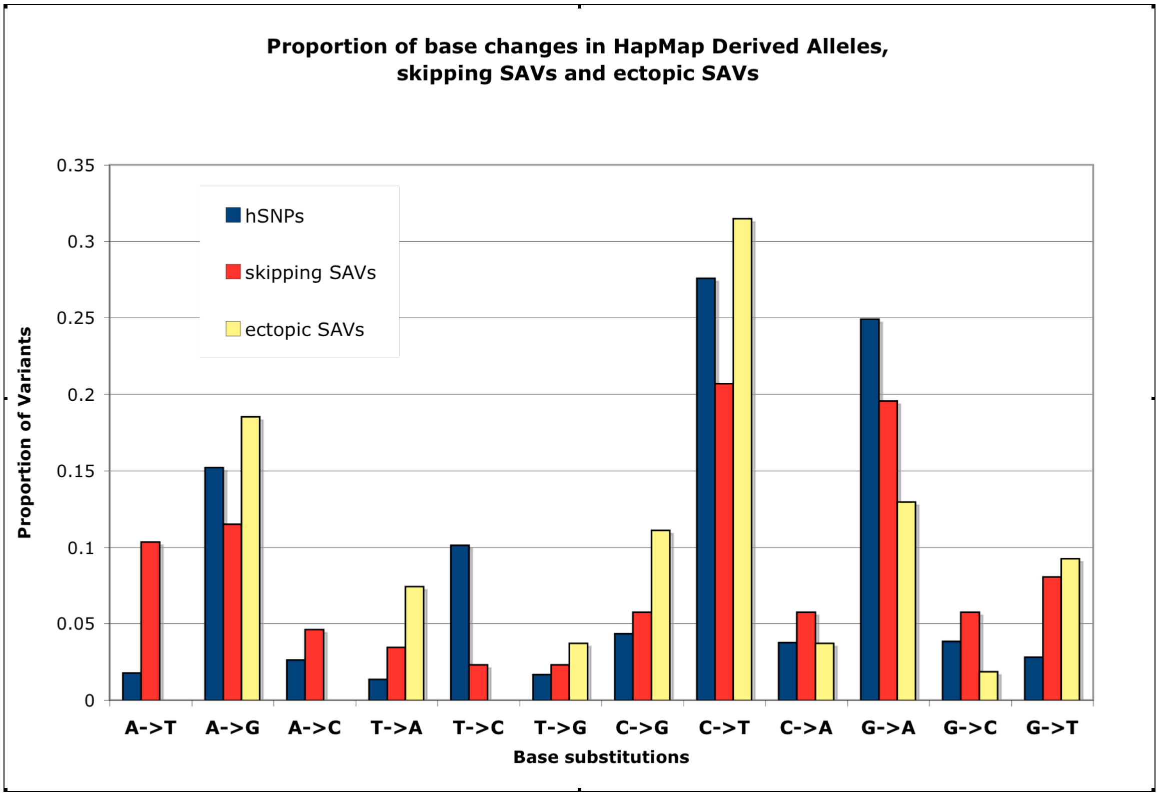

Figure S1. Distribution of nucleotide substitutions types in 15,547 HapMap derived alleles (‘hSNPs’), 87 exon skipping SAVs (‘skipping SAVs’) and 54 SAVs that create a *de novo* ectopic splice site (‘ectopic SAVs’). Base substitutions in hSNPs were used a background nucleotide substitution rates in calculating an expected distribution of ESE/ESS changes of the NI-ESR hexamer set (Figure 3). Significant differences in distributions between skipping SAVs and hSNPs (such as that seen in A->T, T->C and G->T) and ectopic SAVs (A->T, A->C, T->A, C->G) while potentially of biological interest, should be treated with caution due to small number of skipping and ectopic SAVs and large discrepancy in dataset size between these sets and HapMap SNPs.
